# Factors facilitating or hindering the use of antibiotic-sparing treatment strategies in women with uncomplicated urinary tract infections: a scoping review

**DOI:** 10.1007/s15010-025-02635-4

**Published:** 2025-09-10

**Authors:** Andreas Plate, Stefania Di Gangi, Robin Baumann, Oliver Senn, Stefan Neuner-Jehle

**Affiliations:** Institute of Primary Care, University of Zurich and University Hospital Zurich, Pestalozzistrasse 24, Zurich, 8091 Switzerland

**Keywords:** Urinary tract infection, Delayed prescribing, Antibiotic therapy, Facilitator, Barrier, Scoping review

## Abstract

**Purpose:**

Antibiotic-sparing treatment (ASPT) strategies, such as delayed prescribing and symptomatic treatment, are promising to reduce antimicrobial consumption (AMC) in patients with uncomplicated urinary tract infections (uUTI). The aim of this scoping review was to identify literature reporting on factors that may act as barriers and facilitators to the use of ASPT in order to improve implementation.

**Methods:**

MEDLINE (Ovid), Embase, the Cochrane Database, Google Scholar, Proquest Dissertations and Theses, the Clinical Trials Gov Registry and the ICTRP WHO Registry were searched for evidence of health care professionals and/or patients exposed to ASPT in the context of uUTI. We included evidence published between 2000 and 2024, from high-income countries and in any language. Identified factors were grouped into themes and categorized as facilitators or barriers.

**Results:**

A total of 6543 unique records were screened for eligibility and 108 records were included in the review. Most evidence was from original research (*n* = 50, 46.3%) or reviews (*n* = 46, 42.6%). We identified AMC, clinical outcomes, healthcare utilisation, and patient- or prescriber-related factors as main themes. The main facilitator was the expectation of reduced AMC, while prolonged symptom duration and increased risk of disease progression were identified as main barriers.

**Conclusion:**

The clinical management of uUTIs is shaped by factors that can facilitate or hinder ASPT use. This scoping review identified key factors and provided a basis for future research in the area of patient-provider decision making for ASPT, with the ultimate goal to inform targeted interventions and promote wider implementation of ASPT.

**Supplementary Information:**

The online version contains supplementary material available at 10.1007/s15010-025-02635-4.

## Background

Antimicrobial resistance (AMR) is a major threat to public health. The main driver of increasing AMR rates is the use of antibiotics [1]. Interventions to promote prudent use of antibiotics are therefore of great importance. The majority of antibiotics are prescribed in the outpatient setting, with primary care being the most important prescribing group [2]. Apart from respiratory tract infections (RTIs), acute uncomplicated lower urinary tract infections (uUTI) in women are the most common reason for prescribing antibiotics [3–5].

Although uUTI is usually a self-limiting condition, antibiotics are the standard treatment for these patients up to now [6]. Treatment is primarily aimed at reducing symptoms, as progression to upper urinary tract infection is rare even in the absence of treatment [6–8]. In recent years, with increasing rates of AMR, more attention has been paid to attempts to treat uUTIs primarily symptomatically. The main aim of these alternative strategies is to safely (i.e. avoiding complication or progression) reduce the use of antibiotics while still providing symptoms relief [9].

Antibiotic-sparing treatment (ASPT) strategies, such as delayed prescription of antibiotics and/or symptomatic treatment, have been successfully tested in several trials. As a result, the use of ASPT strategies is recommended in an increasing number of clinical guidelines [10–13]. However, the use of these ASPT strategies also has negative aspects, such as prolonged symptom duration and a slightly increased risk of upper urinary tract infection [9, 14, 15]. The available evidence shows that a large proportion of patients with uUTI are still treated with immediate antibiotics [16]. In order to plan interventions to improve the implementation of ASPT strategies, it is important to know what factors, e.g. attitudes, experiences, barriers or facilitators, influence the use of ASPT strategies in patients with uUTI. To our knowledge, such an overview does not exist. Therefore, the aim of this scoping review was to map the current evidence on factors that may affect the decision to use ASPT.

## Methods

The review was conducted according to the methodology for scoping reviews proposed by the Joanna Briggs Institute (JBI) [17]. The protocol of the scoping review was published in advance [18] and the scoping review was registered at the Open Science Framework Registry [19].

Review questions: The main question is: What are the factors, e.g. facilitators or barriers, affecting decisions to use ASPT strategies in women with uUTI? We also aimed to identify factors related to provider’s profession (e.g., general practitioners (GPs), pharmacists, other healthcare professionals (HCP)) and to identify knowledge gaps regarding attitudes toward ASPT.

Participants: We considered evidence including HCP (e.g. physicians, nurses, pharmacists) and/or patients exposed to the concept of ASPT in the context of uUTI. Due to the heterogeneous definitions of uUTI [20], we did not create a separate definition for this scoping review. Evidence was included if uUTI was defined according to the common understanding of uUTI: this usually refers to adult, non-pregnant women with no known anatomical or functional limitations of the urinary tract [13]. Therefore, evidence from men, children/adolescents, people > 65 years of age or pregnant women was not included.

Concept: We included evidence on ASPT that reported on factors, e.g. outcomes, that may act as facilitators or barriers, or both, in the decision to use ASPT. These factors included experiences, attitudes, clinical outcomes or beliefs expressed by HCP or patients in the use of ASPT. ASPT strategies were defined as (1) symptomatic treatments, such as painkillers or herbal remedies, recommended by a healthcare professional, (2) delayed prescription of antibiotics, and (3) combinations of delayed prescribing and symptomatic treatments.

Context: We considered evidence from the outpatient setting, such as primary care, general practice, family medicine practices, emergency rooms, or pharmacies. We excluded evidence from inpatient settings. We excluded evidence from low- and middle-income countries (LMIC), as defined by the World Bank Country and Lending Groups classification in 2024 [21].

Type of sources: For this review we considered experimental and quasi-experimental study designs, observational studies, qualitative and mixed-methods studies, reviews, and guidelines published in peer-reviews journals. In addition, we considered sources of scholarly literature that are not commercially published, commonly referred to as ‘grey literature’. These include conference abstracts, theses or dissertations, government or policy documents, and books.

Search strategy: The search strategy was developed in collaboration with an experienced librarian from the University Library of Zurich and was based on the following four concepts: UTI, antibiotics, antibiotic-sparing treatments and facilitators and barriers. The search terms used can be found in the Supplementary Appendix. The following databases were searched: MEDLINE (Ovid), Embase, the Cochrane Database, Google Scholar, Proquest Dissertations and Theses, Clinical Trials Gov Registry and the ICTRP WHO Registry. An initial search including MEDLINE (Ovid), Embase and the Cochrane Database was conducted on 18/03/2024 and a second (update) search, including non-commercially published scholarly sources, was conducted on 20/12/2024. The Google Scholar search was performed on 18/12/2024. The search itself was carried out by the librarian. We searched for literature in all languages from 2000 onwards. The search strategy is available as supplementary material (Supplemental Tables 1–7).

Selection of evidence: After removing duplicates all abstracts were screened independently by three authors (AP, SD, RB). Where necessary, the full text was reviewed for inclusion by two or three authors. Disagreements were solved by discussion, including a team member not involved in the title/abstract screening (SNJ). A list of sources excluded after full-text review is provided in Supplemental Table 8.

Data extraction and analysis: A self-developed tool was used for data extraction. The tool was pilot tested with ten sources. Data extractions are provided in Supplemental Table 9. All data were extracted by two authors. To answer the research questions, we conducted a content analysis using an inductive approach. All identified factors that could influence the use of ASPT were categorized into themes and, where appropriate, sub-themes. The categorization was discussed within the research team. Characteristics of sources of evidence were reported in tabular format. Common frequencies regarding the number of evidence sources that used a particular method (observational, experimental, qualitative…) and the location / country / context where the evidence source were conducted was reported.

The results of this scoping review are reported in line with the Preferred Reporting Items for Systematic Reviews and Meta-Analyses extension for Scoping Reviews (PRISMA-ScR) [22]. The checklist is provided in the supplemental (Supplemental Table 10).

## Results

**Fig. 1:**
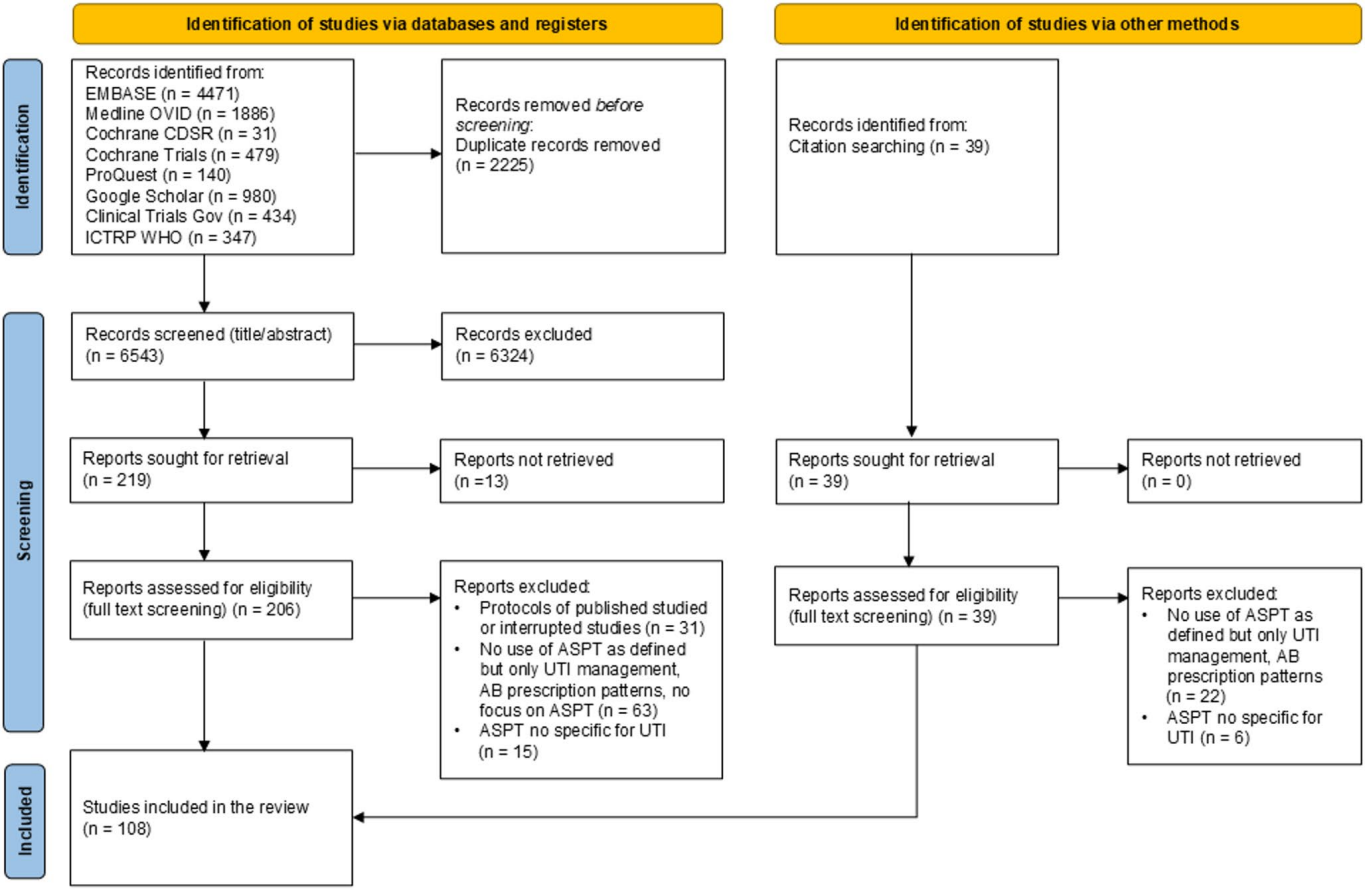
PRISMA Flow Diagram. Abbreviations: AB: antibiotic; ASPT: Antibiotic-sparing treatment; UTI: Urinary tract infection

The search yielded 8768 records, of which 2225 were duplicates. In addition, a further 42 records identified from the reference lists were checked for eligibility. After title/abstract and full text screening, 108 records were included in the final review (Fig. 1). These records included 50 original research studies, 46 reviews and 12 other reports.

### Characteristics of included records

**Fig. 2:**
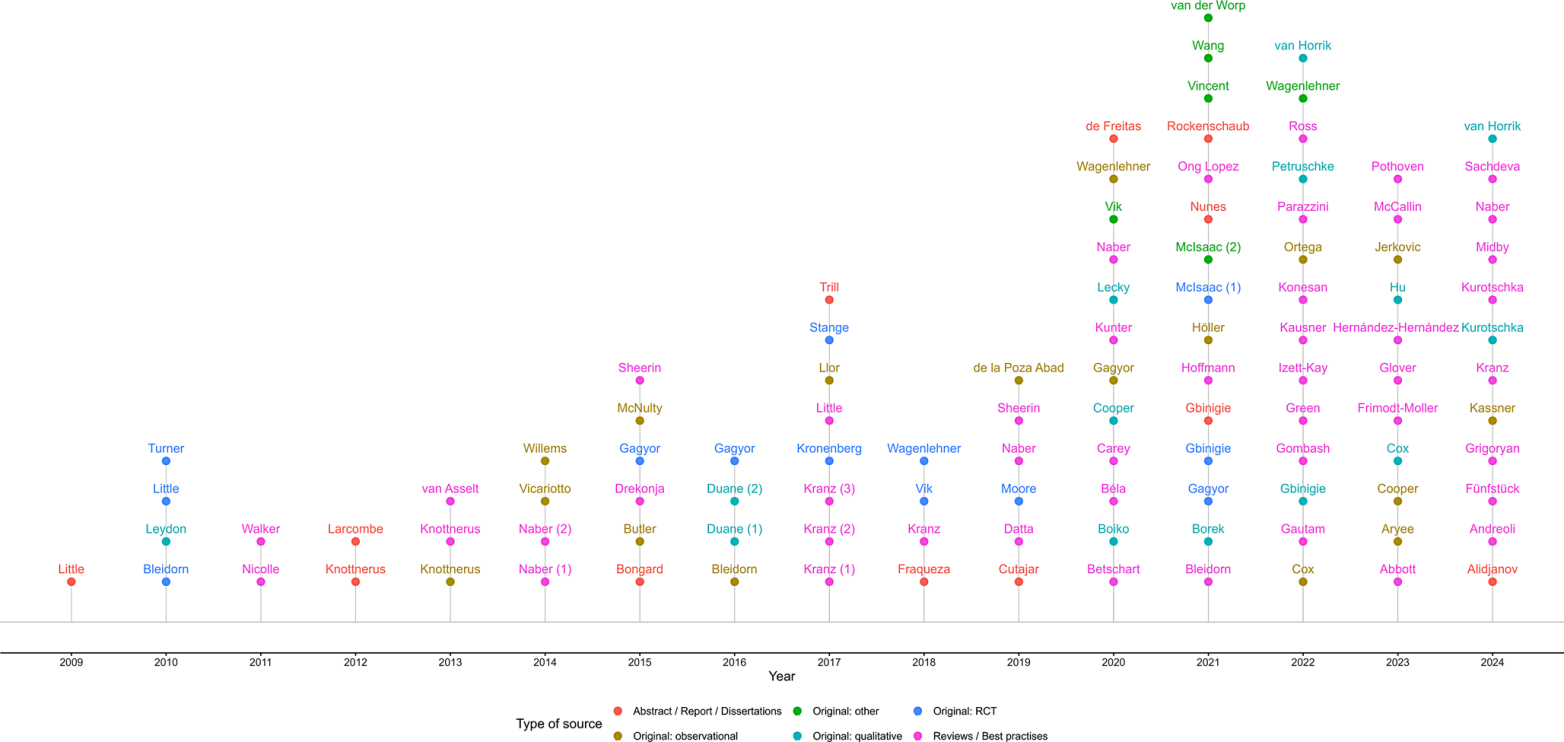
Timeline of included records. The name of the first author and the year of publication are shown

A detailed overview of the included records is presented in Table 1 and Supplementary Table 9. Most publications were in English (91, 84.3%), with most of the original research studies originating from Europe (94%). Almost all studies were conducted in general practice or primary care settings, with only one study specifically set in a pharmacy context. There has been an increase in the number of studies and reviews focusing on ASPT, particularly between 2020 and 2024 (Fig. 2). Specifically, there has been an increase in the publication of review articles, including systematic reviews and best practice recommendations, addressing ASPT. The focus of the original studies was: pain medication [23–30], herbal treatments [31–43], delay prescribing of antibiotics [44–54] or a combination of both symptomatic treatments and delay prescribing [55–71].

**Table 1:** Basic characteristics of included studies

Study type	Number of included studies	Language	Setting	Country	References
Original research					
Cohort	9	English (*n* = 8), German (*n* = 1)	GP/PC (*n* = 6), ambulatory/outpatient (*n* = 3)	Germany (*n* = 5), Netherlands (*n* = 1), UK (*n* = 1), Switzerland (*n* = 1), Italy (*n* = 1)	[24, 27, 34, 36, 38, 40, 43, 44, 47]
Cross sectional	8	English (*n* = 8)	GP/PC (*n* = 4), population/community (*n* = 2), ambulatory/outpatient (*n* = 2)	UK (*n* = 3), Netherlands (*n* = 2), Spain (*n* = 2), Croatia (*n* = 1)	[45, 46, 50, 51, 54, 57, 59, 62]
Intervention	1	English (*n* = 1)	GP/PC (*n* = 1)	Canada (*n* = 1)	[66]
Mixed	3	English (*n* = 3)	GP/PC (*n* = 1), ambulatory/outpatient (*n* = 1), population/community (*n* = 1)	France (*n* = 1), USA (*n* = 1), Netherlands (*n* = 1)	[68, 70, 71]
Qualitative	14	English (*n* = 13), German (*n* = 1)	GP/PC (*n* = 13), pharma (*n* = 1)	UK (*n* = 7), Netherlands (*n* = 3), Ireland (*n* = 2), Germany (*n* = 2)	[33, 35, 48, 53, 55, 56, 58, 60, 61, 63, 64, 67, 69, 74]
RCT	13	English (*n* = 13)	GP/PC (*n* = 11) ambulatory/outpatient (*n* = 2)	UK (*n* = 4), Germany (*n* = 5), Switzerland (*n* = 1), Canada (*n* = 1), German-Poland-Ukraine (*n* = 1), Norway-Sweden-Denmark (*n* = 1)	[23, 25, 26, 28, 29, 31, 32, 37, 39, 42, 49, 52, 65]
Secondary data analysis	2	English (*n* = 2)	GP/PC (*n* = 1) ambulatory/outpatient (*n* = 1)	Norway-Sweden-Denmark (*n* = 1), Germany (*n* = 1)	[30, 41]
Reviews, Guidelines and Best practice					
Reviews	29	English (*n* = 26), German (*n* = 2), Russian (*n* = 1)	ambulatory/outpatient (*n* = 25) GP/PC (*n* = 4)	Germany (*n* = 8), USA (*n* = 6), UK (*n* = 5), Australia (*n* = 2), International* (*n* = 2), Netherlands (*n* = 1), Denmark (*n* = 1), Spain (*n* = 1), Switzerland (*n* = 1), Canada (*n* = 1), Italy (*n* = 1)	[9, 14, 15, 78, 82, 83, 85–90, 92, 94, 100–107, 109, 119, 122, 123, 128–130]
Guidelines, best practice, commentaries	17	English (*n* = 8), German (*n* = 5), Dutch (*n* = 2), French (*n* = 1), Hungarian (*n* = 1)	ambulatory/outpatient (*n* = 15) GP/PC (*n* = 2)	Germany (*n* = 7), UK (*n* = 2), Netherlands (*n* = 2), Switzerland (*n* = 2), USA (*n* = 1), Australia (*n* = 1), Canada (*n* = 1), Hungary (*n* = 1)	[13, 79–81, 84, 91, 93, 95–99, 103, 108, 110, 120, 124]
Abstracts, Reports and Dissertations					
Abstracts	2	English (*n* = 2)	ambulatory/outpatient (*n* = 2)	Germany (*n* = 1), Malta (*n* = 1)	[77, 111]
Dissertation, thesis	9	English (*n* = 6), Portuguese (*n* = 3)	ambulatory/outpatient (*n* = 4), ambulatory/pharma (*n* = 1), pharma (*n* = 1), GP/PC (*n* = 3)	UK (*n* = 5), Portugal (*n* = 3), Netherlands (*n* = 1)	[72, 73, 75, 76, 112–115, 117]
Report / Monograph	1	English (*n* = 1)	ambulatory/outpatient (*n* = 1)	UK (*n* = 1)	[116]

Abbreviations: GP: General Practice; PC: Primary Care; UK: United Kingdom; RCT: Randomized controlled trial

*Factors affecting treatment decisions: themes and subthemes*: The contextualized study results were assigned to five main themes. All themes and sub-themes, as well as the most important barriers and facilitators, are presented in Table 2.

**Table 2:** Barriers and facilitators

Theme	Sub-theme	Barriers		Facilitators	
			Reference		Reference
Antibiotic consumption				Less AMC compared to immediate antibiotic therapy	[23, 25, 28, 31, 32, 34, 36, 37, 42, 46, 47, 49, 65, 71]
Clinical outcomes^1^	Adverse events	Occurrence of SAE or pyelonephritis	[28, 29, 31, 32, 42]	Occurrence of SAE or pyelonephritis	[23–25, 31, 32, 34, 36–38, 47, 52, 72]
	Microbiological cure	Negative or positive urine cultures at different time points	[28, 29, 39, 42]	Negative or positive urine cultures at different time points	[23]
	Symptom burden	Symptom burden at different time points, clinical cure, duration of symptoms	[25, 28, 29, 31, 32, 37, 39, 42, 49, 71]	Symptom burden at different time points, clinical cure, duration of symptoms	[23, 32, 34, 36, 37, 40, 41, 43, 47, 49]
	Side effects^2^			Gastrointestinal symptoms	[39, 42, 43, 73]
	Relapse	Recurrence of UTI at different time points	[23]	Recurrence of UTI at different time points	[24, 27, 31, 34, 36, 39]
Health care utilisation^1^	Costs	Cost effectiveness, costs of initial visit	[52, 71]	Cost effectiveness, costs of initial visit	[52, 71]
	Re-consultations	Re-consultation rates, time to re-consultation	[23, 28, 31]	Re-consultation rates, time to re-consultation	[37, 38, 49, 52]
	Resource use	Urine samples taken, dipsticks performed, consultation time	[52, 53, 63, 64, 69]	Urine samples taken, dipsticks performed, consultation time	[46, 52, 63]
Patient-related factors	Attitudes	Doubts about ASPT	[60]	Willingness to delay antibiotic treatment	[47, 51, 54, 68]
		Previous UTI / UTI complications	[33, 55, 58–61, 64, 74]	Welcoming ASPT	[48, 53, 70]
		Taste of too much sugar in cranberry juice	[33, 76]	Aversion towards antibiotics	[77]
		Nothing is better than antibiotics for UTI	[53, 75]	Preference for a short antibiotic delay	[33]
		Alternatives are better for prevention than cure	[73]	Self-treatment options	[33, 53, 62]
		Herbal medicines considered as plants	[33]	Previous experience with delay	[46]
		Concerns about the cost of alternative therapies	[63]		
	Knowledge	Not aware about ASPT	[51, 53]	AB is not always necessary for mild symptoms	[33, 62]
		Not linking analgesia with UTI treatment	[33, 54, 58]	Awareness about UTI and ASPT	[51, 59, 69]
		Lack of knowledge about harmless of UTI	[58, 74]	Higher education	[68]
		Lack of knowledge about side effects of antibiotics	[58]		
	Patient & Clinical characteristics	Early consultations of patients	[54, 59]	Symptoms are not severe and no risk for complication	[73]
		Positive dipstick results	[26, 47]	Uncertain diagnosis	[59]
		Severe symptoms/ pain	[45, 60, 69]	Young age	[57]
	Expectations	Receiving a delay antibiotic is not being taken seriously, wanting a cure	[33, 48]	Avoiding side effects of antibiotics	[48, 73]
		Wishing / expecting AB when consulting	[48, 55, 58, 63, 67, 76]	Being informed about alternatives and risks	[53]
		Faster symptom relief with antibiotics	[27, 69, 73, 76]	Being involved in treatment decision-making	[58, 69]
	Experiences	Satisfaction with (prior) antibiotic treatment	[28, 48, 60, 69]	Satisfaction with ASPT	[37, 39, 43, 46]
		Faster / immediate symptom relief taking antibiotics	[27, 68, 76]	Experience of AMR and side effects	[55]
		ASPT unexperienced patients with mixed feelings about ASPT	[55, 60]		
		Lack of communication about treatment options (SDM)	[69]		
	Fears	Fear of progression or worsening of symptoms	[33, 48, 69]	Side effects of antibiotics	[76]
Prescriber-related factors	Attitudes	Delay when waiting for urine culture	[74]	Delay antibiotic before holidays/ weekends	[53, 63]
		Superiority of antibiotics	[53]	Pharmacists are more protocol driven	[73]
		A delay is not a strategy, is not usual	[33, 53, 74]		
	Behaviour	Not providing information about UTI and ASPT	[53, 57]	ASPT in patients with less severe symptoms or if dipstick is negative	[35, 60, 63, 74]
		Routine to give antibiotics in confirmed UTI cases	[61, 69]		
		Not involving patients in treatment	[53]		
		AB in patients with severe symptoms	[55, 67, 69, 74]		
	Expectations	(Private insured) patients expecting AB treatment	[61, 63]		
		Patients expect treatment	[35, 53, 60, 64, 67, 69, 73, 75]		
		UTI consultations in patients in whom self-treatment did not work	[69, 74]		
	Experiences	ASPT less effective	[53]	Not receiving an antibiotic immediately is becoming widely more accepted	[74]
		Unawareness of patients towards ASPT (in UTI)	[74]	Patients feel better with symptomatic treatment and reduce reliance on antibiotics	[60]
				Patient satisfied with ASPT	[46, 75]
				Long term relationship with patients	[63]
	Fears	Fear of progression or worsening of symptoms	[60, 64, 74]		
	Knowledge	Evidence of herbal medicine is scarce	[35]	AMR as justification	[61]

AMC: Antimicrobial consumption; ASPT: Antibiotic sparing treatment; SAE: Serious adverse event; UTI: Urinary tract infection; AMR: Antimicrobial resistance

1: Conflicting results. Some studies report differences in favour for immediate therapy, while some studies reported no difference

2: Side effects of alternative treatments (e.g. herbal medicine, D-mannose)

- Antimicrobial consumption (AMC) emerged as a key theme in the included studies [23, 25, 28, 29, 31, 34, 36, 37, 42, 44, 46, 47, 49, 52, 65, 66, 71]. Most data on AMC came from randomized controlled trials (RCTs) and cohort studies. AMC was usually measured as the proportion of patients who received an antibiotic within a defined period, although these periods varied considerably, from several days to several weeks. The use of ASPT was consistently associated with a reduction in AMC. Accordingly, this outcome, or rather the expectation of reduced AMC, can be seen as a facilitator.
- Within the broader theme of clinical outcomes, five sub-themes were identified: adverse events [23–25, 28, 29, 31, 32, 34, 36–38, 42, 44, 47, 52, 72], microbiological cure [23, 28, 29, 39, 42], symptom burden [23, 25, 28, 29, 31, 32, 34, 36, 37, 39–43, 47, 49, 71], side effects [39, 42, 43, 73] and relapse [23, 24, 27, 31, 34, 36, 39]. The results of these sub-themes were heterogeneous, largely reflecting the variability in study design, particularly between RCTs and cohort studies. RCTs comparing non-steroidal anti-inflammatory drugs (NSAIDs) with antibiotics reported higher rates of serious adverse events (SAEs), including the occurrence of pyelonephritis [28, 29, 31, 42], and greater symptom burden in patients receiving ASPT [25, 28, 29, 49]. In addition, patients in ASPT arms were less likely to achieve microbiological cure, as indicated by persistent positive urine cultures after treatment [28, 29, 39, 42]. In contrast, several cohort studies investigating herbal therapies did not observe increased rates of SAEs or pyelonephritis [34, 36, 38]. Regarding symptom burden, the results were heterogeneous. While some studies found no differences [23, 34, 36, 37, 43, 49], some studies showed that the ASPT strategy was inferior [25, 28, 29, 31, 32, 39, 42, 49].
- Health care utilisation was identified as the third theme: Information on health care utilisation was derived from both qualitative and quantitative research. Within this category, the sub-themes costs [52, 71], re-consultations [23, 28, 31, 37, 38, 49, 52], and resource utilisation [46, 52, 53, 63, 64, 69] were identified. The heterogeneity of the underlying studies led to some conflicting results. On the other hand, lack of time was repeatedly cited as a reason for prescribing antibiotics directly [63, 64, 69].
- Patient-related factors were identified as the fourth theme. Information on patient-related factors was derived from both qualitative and quantitative research. Attitudes [33, 46–48, 51, 53–55, 58–64, 68, 70, 73–77], clinical characteristics [24, 26, 27, 30, 40, 44, 45, 47, 54, 57, 59, 60, 63, 67, 69, 73], expectations [27, 33, 48, 53, 55, 58, 59, 63, 67, 69, 73, 76], experiences [27, 28, 31, 33, 37, 39, 43, 46, 48, 53, 55, 58–64, 68, 69, 76], fears [33, 48, 69, 76], and knowledge [33, 51, 53, 54, 58, 59, 62, 68, 69, 74] emerged as sub-themes. Several important aspects could be identified within the theme. For example, we found evidence that some patients were willing to delay antibiotics [47, 51, 54, 68] or want to be involved in decision making [53, 58]. On the other hand, some patients were unaware of ASPT concepts or did not perceive pain medication as a treatment for UTIs [33, 51, 53, 54, 58]. In addition, some patients with symptoms of UTI seemed to expect to be prescribed antibiotics, as they expected their symptoms to be relieved more quickly with immediate antibiotic treatment [27, 53, 58, 63, 69] or because they had already tried self-care [60–62, 74] or simply waited before consulting a doctor [48, 54]. This was especially true for those with previous experience of UTI [33, 48, 58–60, 64, 68, 69]. We found evidence that patients reported fear of progression or worsening of symptoms [33, 48, 69].
- Prescriber-related factors were identified as the fifth theme. Sub-themes were attitudes [33, 47, 53, 61, 63, 73, 74], behaviour [35, 50, 53, 55, 57, 60, 61, 63, 66, 67, 69, 74], knowledge [35, 61], fears [60, 64, 74], expectations [35, 53, 60, 61, 63, 64, 67, 69, 73–75], and experiences [39, 46, 53, 56, 60, 63, 67, 74, 75]. We found evidence that antibiotic use in confirmed UTI patients was still routine [61, 69] and that ASPT may be used in patients with mild(er) symptoms or in patients with negative dipstick tests [35, 60, 63, 74]. We found multiple evidence that HCP believed that patients in general or patients with private insurance expected antibiotics [35, 53, 60, 61, 64, 67, 69, 73–75]. We found evidence that HCP mentioned faster symptom relief with antibiotics, but also that ASPT was more accepted by patients and patients were satisfied with this type of treatment [39, 46, 63, 67, 74]. Similar to patients, HCP reported fears of a progression or worsening of symptoms [60, 64, 74].

### Reviews, reports, dissertations and abstracts

The identified reviews and guidelines mainly referred to the RCTs that tested the different ASPT strategies [9, 13–15, 78–109]. A common message from these reviews was that the use of ASPT can lead to a reduction in AMC [9, 14, 15, 79, 82–87, 93, 100, 101, 104, 105, 108, 109]. However, this advantage was offset by the longer duration of patient symptoms and a higher rate of pyelonephritis. Some evidence reported that patients were generally open to ASPT [9, 91, 92, 101, 105, 110]. From the non-peer-reviewed evidence, dissertations and abstracts [72, 73, 75–77, 111–117], some of them contributed to the original content from the themes and sub-themes [72, 73, 75–77].

### Knowledge gaps regarding attitudes toward ASPT

Many studies reported individual factors and some of them analysed the treatment decision [53, 63, 64, 69, 70, 74]. However, none of the studies analysed the causal relationship between these factors and the treatment decision and how these factors may influence attitudes towards ASPT.

## Discussion

This scoping review identified literature reporting on factors that may act as barriers or facilitators, or both, to the decision to use ASPT in patients with uUTI. A total of 108 records were included. This scoping review identified factors within five key themes: AMC, clinical outcomes, healthcare utilisation, patient-related factors and prescriber-related factors. Several individual factors were identified as facilitators or barriers, with reduced AMC being the most important facilitator and increased symptom burden and fear of worsening symptoms being the most important barriers.

This scoping review identified a diverse body of literature addressing different aspects of ASPT. Approximately half of the included studies were original research, while the remainder consisted mainly of reviews and overviews. In addition to the classic approach of relieving symptoms with NSAIDs, an increasing number of trials have tested herbal products. As there is insufficient evidence to support traditional herbal products such as cranberry for the acute treatment of UTI [118], these trials could provide a promising basis for further larger trials investigating these alternative therapies. Notably, there has been a marked increase in the number of publications addressing ASPT over time, particularly in the last five years. This growth is especially evident in the emergence of review articles, which predominantly focus on traditional clinical dimensions, such as the balance between AMC and symptom burden or patient safety. Increased attention may also be triggered by the adoption of ASPT recommendations in guidelines [10–13]. However, studies investigating the practical implementation of ASPT remain limited. Given the increasing endorsement of ASPT in clinical guidelines, there is a need for evidence to support its effective implementation in the different healthcare settings.

### Barriers and facilitators

The two main themes identified, AMC and clinical outcomes, emerged early from the evidence. The reduction in AMC was an important outcome in many identified studies. Conversely, ASPT was associated with increased symptom burden and longer recovery times. This basic trade-off, a reduction in AMC for a small increase in symptom burden, is also the main message of the reviews identified in this scoping review [13, 25, 27–29, 42, 47, 49, 76, 87, 93, 96, 98, 99, 119–124]. Regarding AMC, it should be noted that a reduction in AMC is the main desired outcome of ASPT. At the same time, the prospect of achieving this outcome may be key to facilitating the use of ASPT.

However, increased symptom burden does not seem to be a barrier per se. For example, we found evidence on several occasions that patients reported that they expected antibiotic therapy for their symptoms and that fear of infection progression was also a reason for immediate antibiotic therapy. At the same time, there was evidence that patients valued alternative therapies and were willing to try ASPT strategies. This shows that individual factors, such as symptom burden or risk of infection progression, are perceived differently by patients. Patients make different treatment decisions based on their individual perceptions. This means that knowledge of the identified contextualized barriers or facilitators is less important than knowledge of the factors themselves. Exploring these perceptions allows HCPs to respond to patients’ expectations and concerns as they arise in everyday clinical practice. This also highlights that the barrier/facilitator classification in this review should be seen in the context of the individual studies and does not reflect individual patient perception. At the same time, however, it remains unclear how these complex issues interact in decision making, and how they are weighted in each context.

### Implementation challenges and interprofessional aspects

Although ASPT strategies are increasingly endorsed in clinical guidelines, current evidence suggests that the majority of patients still receive immediate antibiotic therapy [74, 86, 125]. This discrepancy is most likely multifactorial. Firstly, there is usually a delay between the publication of guideline recommendations and their adoption in routine clinical practice. Patients seem to be more aware of ASPT strategies in the context of RTI than uUTI, but GPs seem confident that withholding immediate antibiotic therapy is becoming more widely accepted [74]. Secondly, this review highlights numerous barriers to ASPT implementation from both patient and physician perspectives. Notably, many patients self-medicate and only consult a HCP if their symptoms persist or worsen [60–62, 74]. Moreover, individuals seeking medical care may be those experiencing more severe symptoms [48, 54, 57]. Consequently, it is plausible that a substantial proportion of patients who would be appropriate candidates for ASPT may not engage with a HCP until it is too late, relying on self-management or seeking advice from pharmacists instead. It would therefore be of great interest to assess the proportion of patients with uUTIs who formally qualify for an ASPT strategy.

A secondary objective was to identify factors related to the profession of the HCP. In addition to GPs, the pharmacy setting is of particular interest as more and more pharmacies are providing direct healthcare services, such as advice and treatment for patients with uUTIs [126, 127]. However, we could only identify one study that explicitly emerged from the pharmacy setting. As patients receive advice and healthcare services from different professionals, it is important to understand the arguments and factors involved in SDM. Ideally, both physicians and pharmacists would collaborate, addressing the same basic factors and providing similar advice. Further research on this aspect is therefore highly valuable.

### Strengths and limitations

This review provides an up-to-date and comprehensive overview of the factors that may hinder or facilitate the implementation of ASPT in clinical practice. It establishes a basis for future research exploring the specific individual factors and their interplay in clinical decision-making processes. By incorporating a broad spectrum of sources, including non-commercially published scholarly sources, the most prevalent and relevant factors influencing ASPT use have been captured. It is important to note that this review intentionally excluded evidence from LMICs; therefore, the findings may not be generalizable to those settings. Caution should also be applied when interpreting the results, given the considerable heterogeneity among the included studies, particularly the RCTs. These RCTs varied in design and tested different combinations of antibiotics, NSAIDs, and herbal therapies, which limits the generalizability of individual outcomes. Additionally, there is an inherent degree of subjectivity in categorising the identified factors. Depending on the analytical perspective, certain factors could reasonably be classified under different sub-themes. In this scoping review, factors were classified as either facilitators or barriers based on the context provided by the original studies. However, this classification should not be interpreted as definitive or universally applicable to all clinical scenarios. Factors need to be contextualized or individualized in relation to the specific clinical scenario.

## Conclusions

The clinical management of uUTI involves a complex interplay of factors that can either facilitate or impede the use of ASPT strategies, or both. The factors identified in this scoping review provide a valuable basis for future in-depth research into decision-making dynamics between patients and healthcare providers. This research is essential for informing targeted interventions and supporting the wider implementation of ASPT in clinical practice.

## Supplementary Information

Below is the link to the electronic supplementary material.

Supplementary Material 1

## Data Availability

The datasets analysed during the current study are not publicly available but may be available from the corresponding author on reasonable request.

## Abbreviations

AMC: Antimicrobial consumption
AMR: Antimicrobial resistance
ASPT: Antibiotic-sparing treatment
GPs: General practitioners
HCP: Healthcare professionals
JBI: Joanna Briggs Institute
LMIC: Low-and middle-income countries
NSAIDs: Non-steroidal anti-inflammatory drugs
RCTs: Randomized controlled trials
RTIs: Respiratory tract infections
SAEs: Serious adverse events
uUTI: Uncomplicated urinary tract infections
