# Supplementary material for: Factors facilitating or hindering the use of antibiotic-sparing treatment strategies in women with uncomplicated urinary tract infections: a scoping review
Source: Infection. 2025 Sep 10;54(1):127–41. doi: 10.1007/s15010-025-02635-4 (PMC12864286; doi:10.1007/s15010-025-02635-4)
Supplement: Supplementary file 1 — Supplementary Material 1 [file 15010_2025_2635_MOESM1_ESM.pdf]

Online Supplemental

Supplemental table 1: Search strategy Ovid MEDLINE		
Database(s): Ovid MEDLINE(R) Epub Ahead of Print and In-Process, In-Data-Review & Other Non-Indexed Citations and Daily December 19, 2024		
**#**	**Searches**	**Results**
1	Urinary Tract Infections/ or exp Bacteriuria/ or exp Pyuria/ or Cystitis/	56735
2	(bacilluria or bacteruria or bacteriuria or cystiti* or pyuria* or urocystiti* or ((urinary-tract or urine or urologic*) adj6 infection*) or (bladder adj3 (inflammation* or infection*)) or ((purulent or pus) adj3 urine)).ti,ab.	74478
3	1 or 2	95162
4	Anti-Bacterial Agents/ or Anti-Infective Agents/ or exp Anti-Infective Agents, Urinary/	508331
5	(antibiotic* or anti-biotic* or antimicrobial* or anti-microbial* or antibacterial* or anti-bacterial* or antiinfective* or anti-infective*).ti,ab.	704435
6	4 or 5	895305
7	exp Antimicrobial Stewardship/ or exp Time-to-Treatment/	14779
8	(door-to-treatment-time* or onset-to-treatment-time* or symptom-to-treatment-time* or time-to-therap* or time-to-treatment* or time-to-intervention* or ((antibiotic* or anti-biotic* or antimicrobial* or anti-microbial*) adj3 stewardship*) or ((antibiotic* or anti-biotic* or antimicrobial* or anti-microbial* or therap* or treatment* or intervention* or prescrib* or prescription*) adj3 (delay* or postpone*)) or ((antibiotic* or anti-biotic* or antimicrobial* or anti-microbial* or therap* or treatment* or intervention*) adj3 alternative*) or (back-up* adj3 (antibiotic* or anti-biotic* or antimicrobial* or anti-microbial* or prescrib* or prescription*)) or ((reduc* or decreas* or cutback* or diminution* or sparing*) adj3 (antibiotic* or anti-biotic* or antimicrobial* or anti-microbial*)) or ((non-antibiotic* or non-anti-biotic* or non-antimicrobial* or non-anti-microbial* or antibiotic-sparing* or anti-biotic-sparing* or antimicrobial-sparing* or anti-microbial-sparing* or stand-by* or standby* or self-care* or selfcare* or symptomatic*) adj3 (strateg* or therap* or treatment* or intervention*)) or (culture adj3 (antibiotic* or anti-biotic* or antimicrobial* or anti-microbial*))).ti,ab.	224733
9	7 or 8	233943
10	Decision Making/ or exp Decision Making, Shared/ or "Attitude of Health Personnel"/ or "Patient Acceptance of Health Care"/ or Patient Compliance/ or exp Patient Participation/ or exp Patient Satisfaction/ or Education/ or Health Education/ or Patient Education as Topic/ or Knowledge/ or Attitude to Health/ or exp Health Knowledge, Attitudes, Practice/ or risk factors/ or Pyelonephritis/ or exp Pyonephrosis/	1758438

11	(attitude* or accepta* or choice* or choicemaking or decision* or decisionmaking or role* or experience* or practice* or compliance* or engagement* or expectation* or feeling* or involv* or inexperience* or intention* or knowledge* or motivation* or participation* or preference* or satisfaction* or willingness* or understanding* or understood or paradigma* or education* or consider* or risk or risks or benefit* or beneficial* or facilitator* or favour* or favor* or oppos* or perspective* or advice* or advis* or hazard* or recover* or factor* or variable* or pyelonephros* or pyonephros* or uroseps* or pyelonephriti* or (taken adj3 seriously) or (change adj3 (behavior* or behaviour* or practice* or prescrib* or prescription*)) or ((kidney or renal) adj3 infection*) or ((kidney or renal) adj3 (abscess* or carbuncle*)) or (infected adj3 hydronephrosis) or ((pyelonephrotic or pyonephrotic) adj3 kidney) or ((urinary or uro) adj3 seps*) or ((purulent or pus) adj3 urine) or (bladder adj3 inflammation*)).ti,ab.	17431874
12	10 or 11	17767507
13	3 and 6 and 9 and 12	2284
14	13 not (animals not humans).sh.	2236
15	14 not ((exp child/ or exp infant/ or exp adolescent/) not exp adult/)	2024
16	limit 15 to yr="2000 - 2024"	1886
17	(13 not (animals not humans).sh. not ((exp child/ or exp infant/ or exp adolescent/) not exp adult/)) and limit 13 to yr="2000 - 2024"	1886

Supplemental table 2: Search strategy: Embase		
**No.**	**Query**	**Results**
#17	#13 NOT (('animal'/de OR 'animal experiment'/exp OR 'nonhuman'/de) NOT ('human'/exp OR 'human experiment'/de)) NOT (([infant]/lim OR [child]/lim OR [adolescent]/lim) NOT ([adult]/lim OR [aged]/lim)) AND [2000-2024]/py	4471
#16	#15 AND [2000-2024]/py	4471
#15	#14 NOT (([infant]/lim OR [child]/lim OR [adolescent]/lim) NOT ([adult]/lim OR [aged]/lim))	4615
#14	#13 NOT (('animal'/de OR 'animal experiment'/exp OR 'nonhuman'/de) NOT ('human'/exp OR 'human experiment'/de))	5015
#13	#3 AND #6 AND #9 AND #12	5232
#12	#10 OR #11	23028023
#11	attitude*:ti,ab,kw OR accepta*:ti,ab,kw OR choice*:ti,ab,kw OR choicemaking:ti,ab,kw OR decision*:ti,ab,kw OR decisionmaking:ti,ab,kw OR role*:ti,ab,kw OR experience*:ti,ab,kw OR practice*:ti,ab,kw OR compliance*:ti,ab,kw OR	22381262

	engagement*:ti,ab,kw OR expectation*:ti,ab,kw OR feeling*:ti,ab,kw OR involv*:ti,ab,kw OR inexperience*:ti,ab,kw OR intention*:ti,ab,kw OR knowledge*:ti,ab,kw OR motivation*:ti,ab,kw OR participation*:ti,ab,kw OR preference*:ti,ab,kw OR satisfaction*:ti,ab,kw OR willingness*:ti,ab,kw OR understanding*:ti,ab,kw OR understood:ti,ab,kw OR paradigma*:ti,ab,kw OR education*:ti,ab,kw OR consider*:ti,ab,kw OR risk:ti,ab,kw OR risks:ti,ab,kw OR benefit*:ti,ab,kw OR beneficial*:ti,ab,kw OR facilitator*:ti,ab,kw OR favour*:ti,ab,kw OR favor*:ti,ab,kw OR oppos*:ti,ab,kw OR perspective*:ti,ab,kw OR advice*:ti,ab,kw OR advis*:ti,ab,kw OR hazard*:ti,ab,kw OR recover*:ti,ab,kw OR factor*:ti,ab,kw OR variable*:ti,ab,kw OR pyelonephros*:ti,ab,kw OR pyonephros*:ti,ab,kw OR uroseps*:ti,ab,kw OR pyelonephriti*:ti,ab,kw OR ((taken NEAR/3 seriously):ti,ab,kw) OR ((change NEAR/3 (behavior* OR behaviour* OR practice OR prescrib* OR prescription*)):ti,ab,kw) OR (((kidney OR renal) NEAR/3 infection*):ti,ab,kw) OR (((kidney OR renal) NEAR/3 (abscess* OR carbuncle*)):ti,ab,kw) OR ((infected NEAR/3 hydronephrosis):ti,ab,kw) OR (((pyelonephrotic OR pyonephrotic) NEAR/3 kidney):ti,ab,kw) OR (((urinary OR uro) NEAR/3 seps*):ti,ab,kw)	
#10	'decision making'/de OR 'patient decision making'/exp OR 'shared decision making'/exp OR 'health personnel attitude'/de OR 'physician attitude'/exp OR 'patient attitude'/de OR 'patient compliance'/de OR 'patient participation'/exp OR 'patient preference'/de OR 'patient satisfaction'/de OR 'behavior change'/exp OR 'education'/de OR 'health education'/de OR 'patient education'/exp OR 'knowledge'/de OR 'attitude to health'/exp OR 'risk factor'/de OR 'kidney infection'/de OR 'kidney abscess'/de OR 'pyonephrosis'/de OR 'urosepsis'/de OR 'pyelonephritis'/de	3189982
#9	#7 OR #8	339520
#8	'door to treatment time*':ti,ab,kw OR 'onset to treatment time*':ti,ab,kw OR 'symptom to treatment time*':ti,ab,kw OR 'time to therap*':ti,ab,kw OR 'time to treatment*':ti,ab,kw OR 'time to intervention*':ti,ab,kw OR (((antibiotic* OR 'anti biotic*' OR antimicrobial* OR 'anti microbial*') NEAR/3 stewardship*):ti,ab,kw) OR (((antibiotic* OR 'anti biotic*' OR antimicrobial* OR 'anti microbial*' OR therap* OR treatment* OR intervention* OR prescrib* OR prescription*) NEAR/3 (delay* OR postpone*)):ti,ab,kw) OR (((antibiotic* OR 'anti biotic*' OR antimicrobial* OR 'anti microbial*' OR therap* OR treatment* OR intervention*) NEAR/3 alternative*):ti,ab,kw) OR (('back up*' NEAR/3 (antibiotic* OR 'anti biotic*' OR antimicrobial* OR 'anti microbial*' OR prescrib* OR prescription*)):ti,ab,kw) OR (((reduc* OR decreas* OR cutback* OR diminution* OR sparing*) NEAR/3 (antibiotic* OR 'anti biotic*' OR antimicrobial* OR 'anti microbial*')):ti,ab,kw) OR ((('non antibiotic*' OR 'non anti biotic*' OR 'non antimicrobial*' OR 'non anti microbial*' OR 'antibiotic sparing*' OR 'anti biotic sparing*' OR 'antimicrobial sparing*' OR 'anti microbial sparing*' OR 'stand by*' OR standby* OR 'self care*' OR selfcare* OR symptomatic*) NEAR/3 (strateg* OR therap* OR treatment* OR intervention*)):ti,ab,kw) OR ((culture NEAR/3 (antibiotic* OR 'anti biotic*' OR antimicrobial* OR 'anti microbial*')):ti,ab,kw)	304627
#7	'antimicrobial stewardship'/exp OR 'therapy delay'/exp OR 'time to treatment'/exp	62524
#6	#4 OR #5	1264743
#5	antibiotic*:ti,ab,kw OR 'anti biotic*':ti,ab,kw OR antimicrobial*:ti,ab,kw OR 'anti microbial*':ti,ab,kw OR antibacterial*:ti,ab,kw OR 'anti bacterial*':ti,ab,kw OR antiinfective*:ti,ab,kw OR 'anti infective*':ti,ab,kw	952972
#4	'antibiotic agent'/de OR 'antiinfective agent'/de OR 'antibiotic therapy'/de OR 'urinary tract antiinfective agent'/de	759592
#3	#1 OR #2	200878
#2	bacilluria:ti,ab,kw OR bacteruria:ti,ab,kw OR bacteriuria:ti,ab,kw OR cystiti*:ti,ab,kw OR pyuria*:ti,ab,kw OR urocystiti*:ti,ab,kw OR ((('urinary tract' OR urine OR urologic*) NEAR/6 infection*):ti,ab,kw) OR ((bladder NEAR/3 (inflammation* OR infection*)):ti,ab,kw) OR (((purulent OR pus) NEAR/3 urine):ti,ab,kw)	117078

#1	'urinary tract infection'/de OR 'bacteriuria'/exp OR 'pyuria'/exp OR 'cystitis'/de	169827

Supplemental table 3: Search strategy: Cochrane		
**ID**	**Search**	**Hits**
#1	((bacilluria OR bacteruria OR bacteriuria OR cystiti* OR pyuria* OR urocystiti*) OR ((urinary-tract OR urine OR urologic*) NEAR/6 (infection*)) OR ((bladder) NEAR/3 (inflammation* OR infection*)) OR ((purulent OR pus) NEAR/3 (urine))):ti,ab,kw	13250
#2	(antibiotic* OR anti-biotic* OR antimicrobial* OR anti-microbial* OR antibacterial* OR anti-bacterial* OR antiinfective* OR anti-infective*):ti,ab,kw	57502
#3	((door-to-treatment-time* OR onset-to-treatment-time* OR symptom-to-treatment-time* OR time-to-therap* OR time-to-treatment* OR time-to-intervention*) OR ((antibiotic* OR anti-biotic* OR antimicrobial* OR anti-microbial*) NEAR/3 (stewardship*)) OR ((antibiotic* OR anti-biotic* OR antimicrobial* OR anti-microbial* OR therap* OR treatment* OR intervention* OR prescrib* or prescription*) NEAR/3 (delay* OR postpone*)) OR ((antibiotic* OR anti-biotic* OR antimicrobial* OR anti-microbial* OR therap* OR treatment* OR intervention*) NEAR/3 (alternative*)) OR ((back-up*) NEAR/3 (antibiotic* OR anti-biotic* OR antimicrobial* OR anti-microbial* OR prescrib* OR prescription*)) OR ((reduc* OR decreas* OR cutback* OR diminution* OR sparing*) NEAR/3 (antibiotic* OR anti-biotic* OR antimicrobial* OR anti-microbial*)) OR ((non-antibiotic* OR non-anti-biotic* OR non-antimicrobial* OR non-anti-microbial* OR antibiotic-sparing* OR anti-biotic-sparing* OR antimicrobial-sparing* OR anti-microbial-sparing* OR stand-by* OR standby* OR self-care* OR selfcare* OR symptomatic*) NEAR/3 (strateg* OR therap* OR treatment* OR intervention*)) OR ((culture) NEAR/3 (antibiotic* OR anti-biotic* OR antimicrobial* OR anti-microbial*))):ti,ab,kw	36917
#4	((attitude* OR accepta* OR choice* OR choicemaking OR decision* OR decisionmaking OR role* OR experience* OR practice* OR compliance* OR engagement* OR expectation* OR feeling* OR involv* OR inexperience* OR intention* OR knowledge* OR motivation* OR participation* OR preference* OR satisfaction* OR willingness* OR understanding* OR understood OR paradigma* OR education* OR consider* OR risk OR risks OR benefit* OR beneficial* OR facilitator* OR favour* OR favor* OR oppos* OR perspective* OR advice* OR advis* OR hazard* OR recover* OR factor* OR variable* OR pyelonephros* OR pyonephros* OR uroseps* OR pyelonephriti*) OR ((taken) NEAR/3 (seriously)) OR ((change) NEAR/3 (behavior* OR behaviour* OR practice OR prescrib* OR prescription*)) OR ((kidney OR renal) NEAR/3 (infection*)) OR ((kidney OR renal) NEAR/3 (abscess* OR carbuncle*)) OR ((infected) NEAR/3 (hydronephrosis)) OR ((pyelonephrotic OR pyonephrotic) NEAR/3 (kidney)) OR ((urinary OR uro) NEAR/3 (seps*))):ti,ab,kw	1274249
#5	#1 AND #2 AND #3 AND #4	540
#6	#1 AND #2 AND #3 AND #4 with Cochrane Library publication date Between Jan 2000 and Dec 2024	510

Supplemental table 4: Search strategy: Google scholar	Results

Full Text Search String	
“urinary|tract|urological|bladder infection|infections”|cystitis Stewardship|non-antibiotic|”delay|delayed|postpone|postponed antibiotic|antibiotics”|”antibiotic alternative|sparing|reduction|standby|” attitude|acceptance|experience|expectations|factors	980

Supplemental table 5: Search strategy: Dissertations & Thesis Proquest	
**Column1**	**Column2**
Search Strategy	
Set#	S1
Searched for	TIAB((bacilluria OR bacteruria OR bacteriuria OR cystiti* OR pyuria* OR urocystiti*) OR ((urinary-tract OR urine OR urologic*) NEAR/6 (infection*)) OR ((bladder) NEAR/3 (inflammation* OR infection*)) OR ((purulent OR pus) NEAR/3 (urine))) OR DISKW((bacilluria OR bacteruria OR bacteriuria OR cystiti* OR pyuria* OR urocystiti*) OR ((urinary-tract OR urine OR urologic*) NEAR/6 (infection*)) OR ((bladder) NEAR/3 (inflammation* OR infection*)) OR ((purulent OR pus) NEAR/3 (urine)))
Databases	ProQuest Dissertations & Theses A&I
Results	2467
Set#	S2
Searched for	TIAB(antibiotic* OR anti-biotic* OR antimicrobial* OR anti-microbial* OR antibacterial* OR anti-bacterial* OR antiinfective* OR anti-infective*) OR DISKW(antibiotic* OR anti-biotic* OR antimicrobial* OR anti-microbial* OR antibacterial* OR anti-bacterial* OR antiinfective* OR anti-infective*)
Databases	ProQuest Dissertations & Theses A&I
Results	41331
Set#	S3

Searched for	TIAB((door-to-treatment-time* OR onset-to-treatment-time* OR symptom-to-treatment-time* OR time-to-therap* OR time-to-treatment* OR time-to-intervention*) OR ((antibiotic* OR anti-biotic* OR antimicrobial* OR anti-microbial*) NEAR/3 (stewardship*)) OR ((antibiotic* OR anti-biotic* OR antimicrobial* OR anti-microbial* OR therap* OR treatment* OR intervention* OR prescrib* or prescription*) NEAR/3 (delay* OR postpone*)) OR ((antibiotic* OR anti-biotic* OR antimicrobial* OR anti-microbial* OR therap* OR treatment* OR intervention*) NEAR/3 (alternative*)) OR ((back-up*) NEAR/3 (antibiotic* OR anti-biotic* OR antimicrobial* OR anti-microbial* OR prescrib* OR prescription*)) OR ((reduc* OR decreas* OR cutback* OR diminution* OR sparing*) NEAR/3 (antibiotic* OR anti-biotic* OR antimicrobial* OR anti-microbial*)) OR ((non-antibiotic* OR non-anti-biotic* OR non-antimicrobial* OR non-anti-microbial* OR antibiotic-sparing* OR anti-biotic-sparing* OR antimicrobial-sparing* OR anti-microbial-sparing* OR stand-by* OR standby* OR self-care* OR selfcare* OR symptomatic*) NEAR/3 (strateg* OR therap* OR treatment* OR intervention*)) OR ((culture) NEAR/3 (antibiotic* OR anti-biotic* OR antimicrobial* OR anti-microbial*))) OR DISKW((door-to-treatment-time* OR onset-to-treatment-time* OR symptom-to-treatment-time* OR time-to-therap* OR time-to-treatment* OR time-to-intervention*) OR ((antibiotic* OR anti-biotic* OR antimicrobial* OR anti-microbial*) NEAR/3 (stewardship*)) OR ((antibiotic* OR anti-biotic* OR antimicrobial* OR anti-microbial* OR therap* OR treatment* OR intervention* OR prescrib* or prescription*) NEAR/3 (delay* OR postpone*)) OR ((antibiotic* OR anti-biotic* OR antimicrobial* OR anti-microbial* OR therap* OR treatment* OR intervention*) NEAR/3 (alternative*)) OR ((back-up*) NEAR/3 (antibiotic* OR anti-biotic* OR antimicrobial* OR anti-microbial* OR prescrib* OR prescription*)) OR ((reduc* OR decreas* OR cutback* OR diminution* OR sparing*) NEAR/3 (antibiotic* OR anti-biotic* OR antimicrobial* OR anti-microbial*)) OR ((non-antibiotic* OR non-anti-biotic* OR non-antimicrobial* OR non-anti-microbial* OR antibiotic-sparing* OR anti-biotic-sparing* OR antimicrobial-sparing* OR anti-microbial-sparing* OR stand-by* OR standby* OR self-care* OR selfcare* OR symptomatic*) NEAR/3 (strateg* OR therap* OR treatment* OR intervention*)) OR ((culture) NEAR/3 (antibiotic* OR anti-biotic* OR antimicrobial* OR anti-microbial*)))
Databases	ProQuest Dissertations & Theses A&I
Results	14815
Set#	S4

Searched for	TIAB((attitude* OR accepta* OR choice* OR choicemaking OR decision* OR decisionmaking OR role* OR experience* OR practice* OR compliance* OR engagement* OR expectation* OR feeling* OR involv* OR inexperience* OR intention* OR knowledge* OR motivation* OR participation* OR preference* OR satisfaction* OR willingness* OR understanding* OR understood OR paradigma* OR education* OR consider* OR risk OR risks OR benefit* OR beneficial* OR facilitator* OR favour* OR favor* OR oppos* OR perspective* OR advice* OR advis* OR hazard* OR recover* OR factor* OR variable* OR pyelonephros* OR pyonephros* OR uroseps* OR pyelonephriti*) OR ((taken) NEAR/3 (seriously)) OR ((change) NEAR/3 (behavior* OR behaviour* OR practice OR prescrib* OR prescription*)) OR ((kidney OR renal) NEAR/3 (infection*)) OR ((kidney OR renal) NEAR/3 (abscess* OR carbuncle*)) OR ((infected) NEAR/3 (hydronephrosis)) OR ((pyelonephrotic OR pyonephrotic) NEAR/3 (kidney)) OR ((urinary OR uro) NEAR/3 (seps*))) OR DISKW((attitude* OR accepta* OR choice* OR choicemaking OR decision* OR decisionmaking OR role* OR experience* OR practice* OR compliance* OR engagement* OR expectation* OR feeling* OR involv* OR inexperience* OR intention* OR knowledge* OR motivation* OR participation* OR preference* OR satisfaction* OR willingness* OR understanding* OR understood OR paradigma* OR education* OR consider* OR risk OR risks OR benefit* OR beneficial* OR facilitator* OR favour* OR favor* OR oppos* OR perspective* OR advice* OR advis* OR hazard* OR recover* OR factor* OR variable* OR pyelonephros* OR pyonephros* OR uroseps* OR pyelonephriti*) OR ((taken) NEAR/3 (seriously)) OR ((change) NEAR/3 (behavior* OR behaviour* OR practice OR prescrib* OR prescription*)) OR ((kidney OR renal) NEAR/3 (infection*)) OR ((kidney OR renal) NEAR/3 (abscess* OR carbuncle*)) OR ((infected) NEAR/3 (hydronephrosis)) OR ((pyelonephrotic OR pyonephrotic) NEAR/3 (kidney)) OR ((urinary OR uro) NEAR/3 (seps*)))
Databases	ProQuest Dissertations & Theses A&I
Results	3828608
Set#	S5
Searched for	[S1] AND [S2] AND [S3] AND [S4]
Databases	ProQuest Dissertations & Theses A&I
These databases are searched for part of your query.	
Results	141
Set#	S6
Searched for	YR(2000-2024)
Databases	ProQuest Dissertations & Theses A&I
Results	3638312

Set#	S7
Searched for	[S5] AND [S6]
Databases	ProQuest Dissertations & Theses A&I
These databases are searched for part of your query.	
Results	140

Supplemental table 6: Search strategy: Clinical Trials Gov
434 trials found for: bacilluria OR bacteruria OR bacteriuria OR cystitis OR cystitides OR pyuria OR urocystitis OR urocystitides OR urinary-infection OR urinary-infections OR urinary-tract-infection OR urinary-tract-infections OR UTI OR bladder-infection OR bladder-infections OR urine-infection OR urine-infections OR urological-infection OR urological-infections 1 (antibiotic OR antibiotics OR anti-biotic OR anti-biotics OR antimicrobial OR antimicrobials OR anti-microbial OR anti-microbials OR antibacterial OR anti-bacterial OR antiinfective OR antiinfectives OR anti-infective OR anti-infectives OR non-antibiotic OR non-antibiotics)

Supplemental table 7: Search strategy: ICTRP WHO
387 records for 347 trials found for: (bacilluria OR bacteruria OR bacteriuria OR pyuria OR cystiti* OR urocystiti* OR "urinary infection*" OR "urinary tract infection*" OR UTI OR "bladder infection*" OR "urine infection*" OR "urologic* infection*") AND (antibiotic* OR antimicrobial* OR antibacterial* OR antiinfective* OR "anti biotic*" OR "anti microbial*" OR "anti infectiv*" OR "non antibiotic*")

Table S10. List of excluded reports after full-text screening

Reason for exclusion	year	Title	Author	Journal	Type of study
No use of ASPT as defined but only UTI management, AB prescription patterns, no focus on ASPT	2004	Uncomplicated urinary tract infection	M. C. Bishop	EAU Update Series	Review
No use of ASPT as defined but only UTI management, AB prescription patterns, no focus on ASPT	2005	Management of urinary tract infections in female general practice patients	E. Hummers-Pradier, A. M. Ohse, M. Koch, W. R. Heizmann and M. M. Kochen	Family Practice	Original
No use of ASPT as defined but only UTI management, AB prescription patterns, no focus on ASPT	2009	The journey from self-care to GP care: a qualitative interview study of women presenting with symptoms of urinary tract infection	G. M. Leydon, S. Turner, H. Smith and P. Little	British Journal of General Practice	Original
No use of ASPT as defined but only UTI management, AB prescription patterns, no focus on ASPT	2013	Urinary tract infections: current and emerging management strategies	A. E. Barber, J. P. Norton, A. M. Spivak and M. A. Mulvey	Clinical Infectious Diseases	Review
Protocols of published studied or interrupted studies	2014	ATAFUTI - A Trial Investigating Alternative Treatments of Adult Female Urinary Tract Infection	G. B. Euctr	https://trialsearch.who.int/Trial2.aspx?TrialID=EUCTR2013-003327-11-GB	Protocol
No use of ASPT as defined but only UTI management, AB prescription patterns, no focus on	2014	Urinary tract infections	J. Vachek, O. Zakiyanov, V. Adámková and V. Tesař	Kardiologicka Revue	Review
No use of ASPT as defined but only UTI management, AB prescription patterns, no focus on ASPT	2015	A randomized controlled trial of a diagnostic algorithm for symptoms of uncomplicated cystitis at an out-of-hours service	M. Bollestad, N. Grude and M. Lindbaek	Scandinavian Journal of Primary Health Care	Original
No use of ASPT as defined but only UTI management, AB prescription patterns, no focus on ASPT	2015	Urinary tract infections in women	J. Van Schoor	SA Pharmaceutical Journal	Review
ASPT no specific for UTI	2016	Acceptability of antibiotic stewardship measures in primary care	M. Giry, C. Pulcini, C. Rabaud, J. M. Boivin, V. Mauffrey and J. Birge	Medecine et Maladies Infectieuses	Original
No use of ASPT as defined but only UTI management, AB prescription patterns, no focus on ASPT	2016	Use of antimicrobial resistance information and prescribing guidance for management of urinary tract infections: survey of general practitioners in the West Midlands	D. Ironmonger, O. Edeghere, S. Gossain and P. M. Hawkey	BMC Infectious Diseases	Original
No use of ASPT as defined but only UTI management, AB prescription patterns, no focus on	2016	Fighting urinary tract infections with antibiotic and non-antibiotic therapies	L. Peri	Urologia (Treviso)	Review
No use of ASPT as defined but only UTI management, AB prescription patterns, no focus on	2016	Diagnosis, Treatment, and Prevention of Urinary Tract Infection	P. Pietrucha-Dilanchian and T. M. Hooton	Microbiology Spectrum	Review
No use of ASPT as defined but only UTI management, AB prescription patterns, no focus on	2017	UroPathogenic Escherichia coli (UPEC) Infections: Virulence Factors, Bladder Responses, Antibiotic, and Non-antibiotic Antimicrobial Strategies	M. E. Terlizzi, G. Gribaudo and M. E. Maffei	Frontiers in Microbiology	Review
Protocols of published studied or interrupted studies	2018	Reducing antibiotic use for uncomplicated urinary tract infection in general practice by treatment with uva-ursi (REGATTA) - a double-blind, randomized, controlled comparative effectiveness trial	K. Afshar, N. Fleischmann, G. Schmiemann, J. Bleidorn, E. Hummers-Pradier, T. Friede, K. Wegscheider, M. Moore and I. Gagyor	BMC Complementary & Alternative Medicine	Protocol
Protocols of published studied or interrupted studies	2018	Sustainable reduction of antibiotic-induced antimicrobial resistance (ARena) in German ambulatory care: study protocol of a cluster randomised trial	M. Kamradt, P. Kaufmann-Kolle, E. Andres, T. Brand, A. Klingenberg, K. Glassen, R. Pos-Doering, L. Uhlmann, K. Hees, D. Weber, A. Gutscher, V. Wambach, J. Szecsenyi and M. Wensing	Implementation Science	Protocol
No use of ASPT as defined but only UTI management, AB prescription patterns, no focus on	2019	Management of urinary tract infection in women: A practical approach for everyday practice	N. F. Abou Heidar, J. A. Degheili, A. A. Yacoubian and R. B. Khauli	Urology Annals	Review
No use of ASPT as defined but only UTI management, AB prescription patterns, no focus on	2019	Treatment of cystitis by Hungarian general practitioners: A prospective observational study	R. Benko, M. Matuz, Z. Juhasz, J. Bognar, R. Bordas, G. Soos, E. Hajdu and Z. Peto	Frontiers in Pharmacology	Original

No use of ASPT as defined but only UTI management, AB prescription patterns, no focus on	2019	Qualitative Analysis of Primary Care Provider Prescribing Decisions for Urinary Tract Infections	L. Grigoryan, S. Nash, R. Zoorob, G. J. Germanos, M. S. Horsfield, F. M. Khan, L. Martin and B. W. Trautner	Antibiotics	Original
No use of ASPT as defined but only UTI management, AB prescription patterns, no focus on	2019	Lower Urinary Tract Infections: Management, Outcomes and Risk Factors for Antibiotic Re-prescription in Primary Care	M. Pujades-Rodriguez, R. M. West, M. H. Wilcox and J. Sandoe	EClinicalMedicine	Original
No use of ASPT as defined but only UTI management, AB prescription patterns, no focus on	2020	Management of urinary tract infection by early-career general practitioners in Australia	A. Davey, A. Tapley, K. Mulquiney, M. van Driel, A. Fielding, E. Holliday, J. Ball, N. Spike, K. FitzGerald and P. Magin	Journal of Evaluation in Clinical Practice	Original
Protocols of published studied or interrupted studies	2020	Strategies to reduce antibiotic use in women with uncomplicated urinary tract infection in primary care: protocol of a systematic review and meta-analysis including individual patient data	J. Heinz, C. Rover, G. Furaijat, Y. Kausner, E. Hummers, T. Debray, A. D. Hay, S. Heytens, I. Vik, P. Little, M. Moore, B. Stuart, F. Wagenlehner, A. Kronenberg, S. Ferry, T. Monsen, M. Lindbaek, T. Friede and I. Gagyor	BMJ Open	Protocol
Protocols of published studied or interrupted studies	2020	Use of a Share Decision Making Tool in the Care of Acute Cystitis Without Risk of Complication in Primary Care	Nct	https://clinicaltrials.gov/show/NCT04272281	Protocol
No use of ASPT as defined but only UTI management, AB prescription patterns, no focus on	2020	Evaluation of a community pharmacy-led test-and-treat service for women with uncomplicated lower urinary tract infection in England	T. Thornley, C. L. Kirkdale, E. Beech, P. Howard and P. Wilson	JAC-antimicrobial Resistance	Original
No use of ASPT as defined but only UTI management, AB prescription patterns, no focus on	2021	Antimicrobial utilization and stewardship in patients with uncomplicated urinary tract infections managed by pharmacists in the community: A sub-study of the RxOUTMAP trial	N. P. Beahm, D. J. Smyth and R. T. Tsuyuki	Journal of the Association of Medical Microbiology and Infectious Disease Canada = Journal officiel de l'Association Pour La Microbiologie Medicale et Iinfectiologie Canada	Original
No use of ASPT as defined but only UTI management, AB prescription patterns, no focus on	2021	Consultations and antibiotic treatment for urinary tract infections in Norwegian primary care 2006-2015, a registry-based study	L. E. A. Haugom, S. Ruths, K. E. Emberland, K. E. R. Eliassen, G. Rortveit and K. A. Wensaas	BMC Family Practice	Original
No use of ASPT as defined but only UTI management, AB prescription patterns, no focus on	2021	Risk factors for treatment failure in women with uncomplicated lower urinary tract infection	R. Martischang, M. Godycki-Cwirko, A. Kowalczyk, K. Kosiek, A. Turjeman, T. Babich, S. Shiber, L. Leibovici, E. von Dach, S. Harbarth and A. Huttner	PLoS ONE [Electronic Resource]	Original
ASPT no specific for UTI	2022	Information about the natural history of acute infections commonly seen in primary care: a systematic review of clinical practice guidelines	K. P. Boaitey, M. Bakhit, N. Krzyzaniak and T. C. Hoffmann	BMC Infectious Diseases	Original
ASPT no specific for UTI	2022	Perceptions, current practices, and interventions of community pharmacists regarding antimicrobial stewardship: A qualitative	C. Durand, A. Chappuis, E. Douriez, F. Poulain, R. Ahmad, F. X. Lescure and N. Peiffer-Smadja	Journal of the American Pharmacists Association	Original
No use of ASPT as defined but only UTI management, AB prescription patterns, no focus on	2022	Using Large-scale Social Media Analytics to Understand Patient Perspectives About Urinary Tract Infections: Thematic Analysis	G. Gonzalez, K. Vaculik, C. Khalil, Y. Zektser, C. Arnold, C. V. Almario, B. Spiegel and J. Anger	Journal of Medical Internet Research	Original
Not retrieved	2022	WOMEN'S VIEWS ON THE MANAGEMENT AND CAUSE OF URINARY TRACT INFECTION: A QUALITATIVE INTERVIEW STUDY	B. S. N. Mullo, C. A. L. Gómez, I. P. Concepción and A. G. L. Jacome	Journal of Pharmaceutical Negative Results	Original
No use of ASPT as defined but only UTI management, AB prescription patterns, no focus on	2022	Urinary tract infection in adults: diagnosis, management and prevention	B. Y. Ng, M. Datoo, G. Pill, L. Dunsmure and K. D. Othonaiou	Pharmaceutical Journal	Review
Not retrieved	2023	Prescribing and Antibiotic Resistance in General Medicine Patients	Y. Anarase and Y. Anarase	International Journal of Tropical Medicine	Review
No use of ASPT as defined but only UTI management, AB prescription patterns, no focus on	2023	Responding to Urinary Tract Infection Symptoms in England's Community Pharmacies	S. Parekh, K. Hand, L. Xu, V. Roberts, F. Pursey, D. Ashiru-Oredope and D. M. Lecky	Antibiotics	Original
No use of ASPT as defined but only UTI management, AB prescription patterns, no focus on ASPT	2023	GPs' Perspective on a Multimodal Intervention to Enhance Guideline-Adherence in Uncomplicated Urinary Tract Infections: A Qualitative Process Evaluation of the Multicentric RedAres Cluster-Randomised Controlled Trial	A. Schuster, P. Tigges, J. Grune, J. Kraft, A. Greser, I. Gágyor, M. Boehme, T. Eckmanns, A. Klingeberg, A. Maun, A. Menzel, G. Schmiemann, C. Heintze and J. Bleidorn	Antibiotics	Original
No use of ASPT as defined but only UTI management, AB prescription patterns, no focus on	2024	Antibiotic utilization and symptom improvement in a retrospective cohort of women with urinary tract infection symptoms	A. I. Melnyk, N. Meckes, H. M. Zyczynski, P. J. Grosse, M. Guirguis and M. S. Bradley	International Urogynecology Journal	Original
Protocols of published studied or interrupted studies	2009	Symptomatic Therapy of Uncomplicated Lower Urinary Tract Infections in the Ambulatory Setting	Nct	https://clinicaltrials.gov/show/NCT01039545	Protocol
Protocols of published studied or interrupted studies	2012	Treatment of cystitis in adult, non-pregnant women	N. O. Euctr	https://trialsearch.who.int/Trial2.aspx?TrialID=EUCTR2012-002776-14-NO see also Ibuprofen Versus Mecillinam for Uncomplicated Cystitis in Adult, Non-pregnant Women https://clinicaltrials.gov/study/NCT01849926 Published protocol Vik, I., Bollestad, M., Grude, N. et al. Ibuprofen versus mecillinam for uncomplicated cystitis - a randomized controlled trial study protocol. BMC Infect Dis 14, 693 (2014). https://doi.org/10.1186/s12879-014-0693-y	Protocol
Protocols of published studied or interrupted studies	2012	Immediate versus conditional treatment of uncomplicated urinary tract infection - a randomized-controlled comparative effectiveness study in general practices	I. Gagyor, E. Hummers-Pradier, M. M. Kochen, G. Schmiemann, K. Wegscheider and J. Bleidorn	BMC Infectious Diseases	Protocol

No use of ASPT as defined but only UTI management, AB prescription patterns, no focus on	2013	Antibiotic treatment of urinary tract infection by community pharmacists: a cross-sectional study	J. L. Booth, A. B. Mullen, D. A. Thomson, C. Johnstone, S. J. Galbraith, S. M. Bryson and E. M. McGovern	British Journal of General Practice	Original
Protocols of published studied or interrupted studies	2013	Supporting the improvement and management of prescribing for urinary tract infections (SIMPle): protocol for a cluster randomized trial	S. Duane, A. Callan, S. Galvin, A. W. Murphy, C. Domegan, E. O'Shea, M. Cormican, K. Bennett, M. O'Donnell and A. Vellinga	Trials [Electronic Resource] see also http://clinicaltrials.gov/show/NCT01913860	Protocol
No use of ASPT as defined but only UTI management, AB prescription patterns, no focus on	2014	Diagnosis and management of urinary tract infections in the outpatient setting: a review	L. Grigoryan, B. W. Trautner and K. Gupta	Jama	Review
Protocols of published studied or interrupted studies	2015	Alternative treatments of adult female urinary tract infection	Isrctn	https://trialsearch.who.int/Trial2.aspx?TrialID=ISRCTN43397016	Protocol
Not retrieved	2016	Can ibuprofen reduce antibiotic prescriptions for uncomplicated UTIs?		Drug and Therapeutics Bulletin	Review
No use of ASPT as defined but only UTI management, AB prescription patterns, no focus on	2016	Intervention to improve the quality of antimicrobial prescribing for urinary tract infection: a cluster randomized trial	A. Vellinga, S. Galvin, S. Duane, A. Callan, K. Bennett, M. Cormican, C. Domegan and A. W. Murphy	CMAJ	Original
Protocols of published studied or interrupted studies	2017	Are antibiotics really necessary to treat urinary tract infections in women or can support be offered with pain relief?		ISRCTN88111427	Protocol
No use of ASPT as defined but only UTI management, AB prescription patterns, no focus on	2017	Uncomplicated Bacterial Community-Acquired Urinary Tract Infection in Adults	J. Kranz, S. Schmidt, C. Lebert, L. Schneidewind, G. Schmiemann and F. Wagenlehner		Review
Protocols of published studied or interrupted studies	2017	Reducing Antibiotic Use for Uncomplicated Urinary Tract Infection in General Practice by Treatment With Uva Ursi (UU)- a Comparative Effectiveness Trial	see also EUCTR2016-000477-21-DE	Nct see also follow-up 2019 https://clinicaltrials.gov/study/NCT03176563 Observational Study of Patients With Uncomplicated Urinary Tract Infection Treated With Antibiotics or Herbal Medicinal Product (Regatta II)	Protocol
Protocols of published studied or interrupted studies	2017	Uva-ursi extract and ibuprofen as alternative treatments of adult female urinary tract infection (ATAFUTI): study protocol for a randomised controlled trial	J. Trill, C. Simpson, F. Webley, M. Radford, L. Stanton, T. Maishman, A. Galanopoulou, A. Flower, C. Eyles, M. Willcox, A. Hay, G. Griffiths, P. Little, G. Lewith and M. Moore	Trials	Protocol
Not retrieved	2018	Diclofenac inferior to norfloxacin for symptom relief of lower UTIs		Drug and therapeutics bulletin	Review
Protocols of published studied or interrupted studies	2019	Cranberries for urinary tract infection	Isrctn	https://trialsearch.who.int/Trial2.aspx?TrialID=ISRCTN10399299	Protocol
No use of ASPT as defined but only UTI management, AB prescription patterns, no focus on	2019	Re: ibuprofen versus Pivmecillinam for Uncomplicated Urinary Tract Infection in WomendA Double-Blind, Randomized Non-Inferiority Trial	I. Vik, M. Bollestad, N. Grude, A. Baerheim, E. Damsgaard, T. Neumark, L. Bjerrum, G. Cordoba, I. C. Olsen and M. Lindbaek	Journal of urology	Commentary
No use of ASPT as defined but only UTI management, AB prescription patterns, no focus on	2019	Prevention and treatment of uncomplicated lower urinary tract infections in the era of increasing antimicrobial resistance-non-antibiotic approaches: a systemic review	S. Wawrysiuk, K. Naber, T. Rechberger and P. Miotla	Archives of Gynecology & Obstetrics	Review
No use of ASPT as defined but only UTI management, AB prescription patterns, no focus on	2020	Nonantibiotic treatments for urinary cystitis: an update	B. M. Barea, R. Veeratterapillay and C. Harding	Current Opinion in Urology	Review
Protocols of published studied or interrupted studies	2021	Non-steroidal anti-inflammatory drugs for treating symptomatic uncomplicated urinary tract infections in non-pregnant adult women	A. Sachdeva, A. Nambiar, B. P. Rai, R. Veeratterapillay and C. Harding	Cochrane Database of Systematic Reviews	Protocol
ASPT no specific for UTI	2005	Interventions to improve antibiotic prescribing practices in ambulatory care	S. R. Arnold, S. E. Straus	Cochrane Database of Systematic Reviews	Review
ASPT no specific for UTI	2007	Use of the bacteriology laboratory to decrease general practitioners' antibiotic prescribing	I. M. Gould, F.M. Mackenzie, L. Shepherd	European Journal of General Practice	Original
Protocols of published studied or interrupted studies	2017	Reducing antibiotic use for uncomplicated urinary tract infection in general practice by treatment with bearberry leaf extract	Euctr DE	https://trialsearchwhoint/Trial2aspx?TrialID=EUCTR2016-000477-21-DE	Protocol
No use of ASPT as defined but only UTI management, AB prescription patterns, no focus on ASPT	2018	Point-of-care urine culture for managing urinary tract infection in primary care: a randomised controlled trial of clinical and cost-effectiveness	C. C Butler, N. A. Francis, E. Thomas-Jones, M. Longo, M. Wootton, C. Llor, P. Little, M. Moore, J. Bates, T. Pickles, N. Kirby, D. Gillespie, K. Rumsby, C. Brugman, M. Gal, K. Hood, T. Verheij	British Journal of General Practice	Original
Protocols of published studied or interrupted studies	2019	Does cranberry extract reduce antibiotic use for symptoms of acute uncomplicated urinary tract infections (CUTI)? Protocol for a feasibility study	O. Gbinigie, J. Allen, A. M. Boylan, A. Hay, C. Heneghan, M. Moore, N. Williams, C. Butler	Trials [Electronic Resource]	Protocol
No use of ASPT as defined but only UTI management, AB prescription patterns, no focus on	2020	Cranberry Extract for Symptoms of Acute, Uncomplicated Urinary Tract Infection: A Systematic Review	O. A. Gbinigie, E. A. Spencer, C. J. Heneghan, J. J. Lee, C. C. Butler	Antibiotics	Review
No use of ASPT as defined but only UTI management, AB prescription patterns, no focus on	2022	Antibiotic consumption and time to recovery from uncomplicated urinary tract infection: secondary analysis of observational data from a point-of-care test trial	A. Gadalla, H. Wise, D. Farewell, K. Hughes, C. Llor, M. Moore, T. J. Verheij, P. Little, C. C. Butler, N. A. Francis	British Journal of General Practice	Original
No use of ASPT as defined but only UTI management, AB prescription patterns, no focus on	2022	Enhancing opportunistic recruitment and retention in primary care trials: lessons learned from a qualitative study embedded in the Cranberry for Urinary Tract Infection (CUTI) feasibility trial	O. A. Gbinigie, A. M. Boylan, C. C. Butler, C. J. Heneghan, S. Tonkin-Crine	BMC Primary Care	Original

ASPT no specific for UTI	2023	Do family medicine residents optimally prescribe antibiotics for common infectious conditions seen in a primary care setting?	B. Ho, S. Kukan, W. McIsaac	J Assoc Med Microbiol Infect Dis Can.	Original
ASPT no specific for UTI	2023	Is Education Alone Enough to Sustain Improvements of Antimicrobial Stewardship in General Practice in Australia? Results of an Intervention Follow-Up Study	R. Sangwan, A. J. Neels, S. M. Gwini, S. K. Saha, E. Athan	Antibiotics	Original
ASPT no specific for UTI	2023	The National Implementation of a Community Pharmacy Antimicrobial Stewardship Intervention (PAMSI) through the English Pharmacy Quality Scheme 2020 to 2022	C. V. Hayes, S. Parekh, D. M. Lecky, J. Loader, C. Triggs-Hodge, D. Ashiru-Oredope	Antibiotics	Original
ASPT no specific for UTI	2023	Addressing Antimicrobial Stewardship in Primary Care—Developing Patient Information Sheets Using Co-Design Methodology	R. Biezen, S. Ciavarella, J. A. Manski-Nankervis, T. Monaghan, K. Buising	Antibiotics	Original
No use of ASPT as defined but only UTI management, AB prescription patterns, no focus on	2002	The impact of empirical management of acute cystitis on unnecessary antibiotic use	W. J. McIsaac, D. E. Low, A. Biringer, N. Pimlott, M. Evans, R. Glazier	Archives of Internal Medicine	Original
Protocols of published studied or interrupted studies	2007	Antibiotics versus ibuprofen for uncomplicated lower urinary tract infections: a randomised controlled double-blind clinical trial in German general practices	ISRCTN00470468	see also Gágyor, I., Bleidorn, J., Wegscheider, K. et al. Practices, patients and (im)perfect data - feasibility of a randomised controlled clinical drug trial in German general practices. Trials 12, 91 (2011). https://doi.org/10.1186/1745-6215-12-91	Protocol
No use of ASPT as defined but only UTI management, AB prescription patterns, no focus on	2021	Prevalence of and factors associated with the prescription of phytopharmaceuticals in outpatients with urinary tract infections	C. Ehrenberg, M. Kalder	International Journal of Clinical Pharmacology and Therapeutics	Original
No use of ASPT as defined but only UTI management, AB prescription patterns, no focus on	2016	Efficacy and Safety of a Medical Device versus Placebo in the Early Treatment of Patients with Symptoms of Urinary Tract Infection: A Randomized Controlled Trial	A. García-Larrosa, O. Alexe	Clinical Microbiology	Original
No use of ASPT as defined but only UTI management, AB prescription patterns, no focus on ASPT	2019	Barriers to guideline adherence: Identification of barriers to guideline adherence using a survey on the AWMF S3 guideline epidemiology, diagnosis, treatment, and management of uncomplicated bacterial, community-acquired urinary tract infections in adult patients	J. Kranz, D. Schlager, S. Mühlstädt, J. Nagler, F. M. E. Wagenlehner, L. Schneidewind	Urologe	Original
Protocols of published studied or interrupted studies	2024	Dipsticks and point-of-care Microscopy to reduce antibiotic use in women with an uncomplicated Urinary Tract Infection (MicUTI): protocol of a randomised controlled pilot trial in primary care	P. K. Kurotschka, G. Borgulya, E. Bucher, I. Endrich, A. Figueiras, J. Gensichen, A. D. Hay, A. Hapfelmeier, C. Kretzschmann, O. Kurzai, T. T. Lam, O. Massidda, L. Sanftenberg, G. Schmiemann, A. Schneider, A. Simmenroth, S. Stark, L. Warkentin, M. H. Ebell, I. Gàgyor; Bavarian Practice-Based Research Network (BayFoNet).	BMJ Open	Protocol
No use of ASPT as defined but only UTI management, AB prescription patterns, no focus on	2023	Cystitis and Utipro® Plus: Real-World Evidence	K. Kostev and T. Cai	Healthcare	Original
No use of ASPT as defined but only UTI management, AB prescription patterns, no focus on	2021	Naturopathic Management of Urinary Tract Infections: A Retrospective Chart Review	L. Garofalo, H. Zwickey, R. Bradley, D. Hanes	Journal of Alternative and Complementary Medicine	Original
No use of ASPT as defined but only UTI management, AB prescription patterns, no focus on	2018	Acute uncomplicated cystitis: is antibiotic unavoidable?	E. Kulchavenya	Therapeutic Advances in Urology	Review
Not retrieved	2006	The practice guideline 'Urinary-tract infections' (second revision) from the Dutch College of General Practitioners; a response from the perspective of general practice	A. J. Boeke	Nederlands Tijdschrift voor Geneeskunde	Review
ASPT no specific for UTI	2024	Mapping the evidence about the natural history of acute infections commonly seen in primary care and managed with antibiotics: a scoping review	K. P. Boaitey, M. Bakhit, T. C. Hoffmann	BMC Infectious Diseases	Review
No use of ASPT as defined but only UTI management, AB prescription patterns, no focus on	2017	Cranberries for treatment and prophylaxis of urinary tract infections - Really helpful?	M. R. Duran, A. Sönnichsen	Zeitschrift fur Allgemeinmedizin	Review
No use of ASPT as defined but only UTI management, AB prescription patterns, no focus on	2024	'When all you have is a hammer, everything has to be a nail': Managing diagnostic uncertainty in urinary tract infection	E. Kashouris	Sociology of Health & Illness	Review
No use of ASPT as defined but only UTI management, AB prescription patterns, no focus on ASPT	2018	The 2017 Update of the German Clinical Guideline on Epidemiology, Diagnostics, Therapy, Prevention, and Management of Uncomplicated Urinary Tract Infections in Adult Patients: Part 1	J. Kranz, S. Schmidt, C. Lebert, L. Schneidewind, F. Mandraka, M. Kunze, S. Helbig, W. Vahlensieck, K. Naber, G. Schmiemann, F. M. Wagenlehner	Urologia Internationalis	Review
No use of ASPT as defined but only UTI management, AB prescription patterns, no focus on	2024	Indian medicinal plants for combating urinary tract infections	S. Kumar, P. Parikh, H. N. Gupta	Medicinal Plants	Review

No use of ASPT as defined but only UTI management, AB prescription patterns, no focus on	2020	Alternative therapeutic options to antibiotics for the treatment of urinary tract infections	P. Loubet, J. Ranfaing, A. Dinh, C. Dunyach-Remy, L. Bernard, F. Bruyère, J. P. Lavigne, A. Sotto	Frontiers in Microbiology	Review
No use of ASPT as defined but only UTI management, AB prescription patterns, no focus on	2024	An update on alternative therapy for Escherichia coli causing urinary tract infections; a narrative review	S. Nasrollahian, F. Moradi, N. Hadi, S. Ranjbar, R. Ranjbar	Photodiagnosis & Photodynamic Therapy	Review
ASPT no specific for UTI	2022	Contribution of symptomatic, herbal treatment options to antibiotic stewardship and microbiotic health	B. Nausch, C. B. Bittner, M. Höller, D. Abramov-Sommariva, A. Hiergeist, A. Gessner	Antibiotics	Review
Not retrieved	2018	Uncomplicated female urinary tract infections comprehensive therapy	E. A. Pronkin	Urologiia (Moscow, Russia)	Review
No use of ASPT as defined but only UTI management, AB prescription patterns, no focus on	2024	The Diagnosis and Treatment of Adult Urinary Tract Infections in the Emergency Department	R. Redwood, K. C. Claeys	Emergency Medicine Clinics of North America	Review
ASPT no specific for UTI	2022	Exploring antimicrobial stewardship influential interventions on improving antibiotic utilization in outpatient and inpatient settings: a systematic review and meta analysis	A. A. Sadeq, S. S. Hasan, N. AbouKhater, B. R. Conway, A. E. Abdelsalam, J. M. Shamseddine, Z. O. E. Babiker, E. F. Nsutebu, S. E. Bond, M. A. Aldeyab	Antibiotics	Review
Not retrieved	2012	Treatment of uncomplicated urinary tract infection	G. Stein, R. Fünfstück	Medizinische Welt	Review
ASPT no specific for UTI	2024	Antibiotic therapy for the most common infections in outpatient practice	M. Trojánek, M. Štefan, L. Bezdíčková, M. Prokeš, H. Žemličková	Vnitrni Lekarstvi	Review
No use of ASPT as defined but only UTI management, AB prescription patterns, no focus on	2021	The use of complex herbal supplements for the prevention and treatment of urinary tract infections. The analysis of active components	A. Y. Tsukanov, E. V. Matveev, A. I. Nurgalieva	Urologiia (Moscow, Russia)	Review
No use of ASPT as defined but only UTI management, AB prescription patterns, no focus on	2014	Urinary tract infections	F. M. Wagenlehner, A. Pilatz, K. Naber and W. Weidner	Aktuelle Urologie	Review
ASPT no specific for UTI	2018	Beyond behavior? Institutions, interactions and inequalities in the response to antimicrobial resistance	C. Will	Sociology of health & illness	Review
No use of ASPT as defined but only UTI management, AB prescription patterns, no focus on	2024	Treatment of uncomplicated urinary tract infections (uUTI) in outpatients in a health center using integrative medicine methods: A retrospective analysis with future perspectives	J. Biechele, T. Haeck and D. Krueerke	Swiss Medical Weekly	Abstract
No use of ASPT as defined but only UTI management, AB prescription patterns, no focus on	2023	Women's satisfaction with the management of urinary tract infections during the COVID-19 pandemic: a qualitative analysis of free-text comments from a national survey in England	A. Dababneh, L. Sanyaolu, H. Ahmed, D. Alagiyawanna, D. Lecky and E. Cooper	JAC-Antimicrobial Resistance	Abstract
No use of ASPT as defined but only UTI management, AB prescription patterns, no focus on	2017	Alternative therapy for acute uncomplicated cystitis	E. Kulchavenya, S. Shevchenko, E. Brizhatyuk	European Urology, Supplements	Abstract
No use of ASPT as defined but only UTI management, AB prescription patterns, no focus on ASPT	2023	I THINK THAT (...) A PART OF SELF-CARE IS BEING ABLE TO SEEK HELP AND GET IT WHEN YOU NEED IT THE MOST.' RESULTS FROM A QUALITATIVE STUDY ON ENABLERS AND BARRIERS TO SELF-CARE OF URINARY TRACT INFECTIONS EXPERIENCED BY WOMEN LIVING IN LOW-INCOME HOUSEHOLDS AND WOMEN FROM MINORITY ETHNIC BACKGROUNDS	A. Pacho, R. E. Glover, H. Bennani, M. Al-Haboubi and N. Mays	Journal of Epidemiology and Community Health	Abstract
No use of ASPT as defined but only UTI management, AB prescription patterns, no focus on	2023	Preventing and managing urinary tract infections: using thematic analysis to explore interventions and strategies implemented by NHS commissioning organizations in English primary care, 2017-22	E. Sides, D. Lecky, E. Taborn, L. O'Neill and E. Cooper	JAC-Antimicrobial Resistance	Abstract
No use of ASPT as defined but only UTI management, AB prescription patterns, no focus on	2013	Non-antibiotic herbal therapy of uncomplicated lower urinary tract infection in women-a pilot study	K. Naber, H. Steindl, D. Abramov-Sommariva, H. Eskoetter	Planta Medica	Abstract
No use of ASPT as defined but only UTI management, AB prescription patterns, no focus on	2016	Core elements of outpatient antibiotic stewardship - MMWR. Recommendations and Reports	G. Sanchez		Pre-print
No use of ASPT as defined but only UTI management, AB prescription patterns, no focus on ASPT	2014	Aplicação de Produtos Derivados do Arando Vermelho no Tratamento e Prevenção de Infeções Urinárias: Experiência Profissionalizante na Vertente de Farmácia Comunitária, Hospitalar e Investigação	S. C. Grilo	Universidade da Beira Interior (Portugal)	Thesis
ASPT no specific for UTI	2009	The role of probiotics for the prevention and treatment of infectious disease	N. Safdar	The University of Wisconsin - Madison	Thesis
No use of ASPT as defined but only UTI management, AB prescription patterns, no focus on	2019	A Systematic Review on the Efficacy of Cranberry-Derived Proanthocyanidins in Urinary Tract Infections	J. Shennan	University of Johannesburg (South Africa)	Thesis

No use of ASPT as defined but only UTI management, AB prescription patterns, no focus on	2021	Relatório de Estágio e Monografia Intitulada "Atividade Antibacteriana do Arando Vermelho Face a Infeções do Trato Urinário Causadas por Escherichia Coli"	S. M. H. Silvestre	Universidade de Coimbra (Portugal)	Thesis
No use of ASPT as defined but only UTI management, AB prescription patterns, no focus on	2023	Fitoterapia no Tratamento das Infeções Urinárias	A. C. R. Baptista	Universidade de Lisboa (Portugal)	Thesis
Protocols of published studied or interrupted studies	2020	A Proof-of-concept, Feasibility Study to Investigate the Effect of Cranberry and Quillaia Extract Type II on Symptoms in Women With Uncomplicated Urinary Tract Infection and to Study the Modulation of the Urinary and Gut Microbiome Pre- and Post-antibiotic Use [Internet]			Protocol
Protocols of published studied or interrupted studies	2020	Utilisation d'un Outil de décision médicale partagée Dans la Prise en Charge Des Cystites Aigues Sans Risque de Complication en médecine générale : Comparaison de la Consommation d'Antibiotique Entre Deux Groupes randomisés en Cluster [Internet]			Protocol
Protocols of published studied or interrupted studies	2021	A double-blind, randomised, multi-centre, controlled clinical trial to compare D-mannose versus antibiotic in the treatment of acute uncomplicated lower urinary tract infections in female patients		EUCTR2021-003466-12-DE	Protocol
Protocols of published studied or interrupted studies	2021	BeaRberry in the Treatment of Acute UncoMplIcated Cystitis (BRUMI)- Protocol of a Multicentre, Randomized Double-Blind Clinical Trial			Protocol
Protocols of published studied or interrupted studies	2023	NSW Government-Sponsored Clinical Trial: Management of Urinary Tract Infections by Community Pharmacists		ACTRN12623000882628	Protocol
Protocols of published studied or interrupted studies	2020	Reduction of antibiotic resistance in uncomplicated urinary tract infections by treatment according to national guidelines in the ambulatory care		DRKS00020389	Protocol
Protocols of published studied or interrupted studies	2018	The Development and Testing of a Scaling Strategy for a Community-Based Primary Care Antimicrobial Stewardship Program		NCT03517215	Protocol
Protocols of published studied or interrupted studies	2020	Cranberry and Quillaja on Symptoms of Uncomplicated UTI		NCT04496726	Protocol
Protocols of published studied or interrupted studies	2024			Piraux, A., Parot-Schinkel, E., Hamel, JF. et al. Efficacy of a pharmacist care protocol to manage uncomplicated female cystitis in community pharmacies: an open-label, multicenter, randomized, controlled, cluster study: the PharmaCyst' protocol. Trials 25, 654 (2024)	Protocol
Protocols of published studied or interrupted studies	2015	A Double-blind, Controlled, Parallel-group, Randomized, Multicenter Clinical Trial to Assess the Efficacy and Safety of a Herbal Drug Containing Centaury, Lovage Root and Rosemary Leaf (CLR) in Comparison to Fosfomycin Trometamol for the Treatment of Acute Lower Uncomplicated Urinary Tract Infections (uUTIs) in			Protocol
Protocols of published studied or interrupted studies	2018	The Development and Testing of a Scaling Strategy for a Community-Based Primary Care Antimicrobial Stewardship Program (PC-ASP 2)			Protocol
Protocols of published studied or interrupted studies	2010	This study aims to find out how effective and save Angocin Anti-Infekt N is in treatment of acute uncomplicated cystitis in contrast to standard antibiotics.		EUCTR2010-022096-54-DE	Protocol
Not retrieved	2023	Cystitis-Updates and Challenges: Updates and Challenges	G. Palleschi and A. Cardi	IntechOpen	Book
Not retrieved	2020	Urinary Tract Infection (Chapter in Obstetrics and Gynecology)	A. Peacher and A. Hardart	Rhoda Sperling	Book
Not retrieved	2023	Antibiotic Stewardship In Geriatric Medicine	P. D. Sloane, C. E. Kistler	Springer, Cham	Book
Not retrieved	2023	Treatment of infectious urinary tract disease	J. Weese, A. Defarges	CABI Digital Library	Book
Not retrieved	2023	Antimicrobial stewardship in the outpatient setting (Chapter in Antimicrobial Stewardship in Non-Traditional Settings)	M. Zmarlicka, J. Chin, G. Vazquez	Springer, Cham	Book
Not retrieved	2024	Urinary Tract Infection (Chapter in Common Cases in Women's Primary Care Clinics)	S. Zuo and M. Bradley	Springer, Cham	Book
No use of ASPT as defined but only UTI management, AB prescription patterns, no focus on	2002	Randomised controlled trial of nitrofurantoin versus placebo in the treatment of uncomplicated urinary tract infection in adult women	T C M Christiaens, M De Meyere, G Verschraegen, W Peersman, S Heytens and J M De Maeseneer	Bristish Journal of General Practice	Original
No use of ASPT as defined but only UTI management, AB prescription patterns, no focus on	2003	The current management strategies for community-acquired urinary tract infection	T. M. Hooton	Infectious Disease Clinics of North America	Review
No use of ASPT as defined but only UTI management, AB prescription patterns, no focus on	2004	The natural course of uncomplicated lower urinary tract infection in women illustrated by a randomized placebo controlled study	S. A. Ferry, S. E. Holm, H. Stenlund, R. Lundholm, T. J. Monsen	Scandinavian Journal of Infectious Diseases	Original

No use of ASPT as defined but only UTI management, AB prescription patterns, no focus on	2005	Response to antibiotics of women with symptoms of urinary tract infection but negative dipstick urine test results: double blind randomised controlled trial	D. Richards, L. Toop, S. Chambers, L. Fletcher	The BMJ	Original
No use of ASPT as defined but only UTI management, AB prescription patterns, no focus on	2007	Clinical and bacteriological outcome of different doses and duration of pivmecillinam compared with placebo therapy of uncomplicated lower urinary tract infection in women: The LUTIW project	S. A. Ferry, S. E. Holm, H. Stenlund, R. Lundholm, T. J. Monsen	Scandinavian Journal of Primary Health Care	Original
No use of ASPT as defined but only UTI management, AB prescription patterns, no focus on	2007	Differences in physician and patient perceptions of uncomplicated UTI symptom severity:understanding the communication gap	F. W. Platt, K. N. Keating	International Journal of Clinical Practice	Original
No use of ASPT as defined but only UTI management, AB prescription patterns, no focus on	2009	Antibiotics versus placebo in the treatment of women with uncomplicated cystitis: A meta-analysis of randomized controlled trials	M. E. Falagas, I. K. Kotsantis, E. K. Vouloumanou, P. I. Rafailidis	Journal of Infection	Review
No use of ASPT as defined but only UTI management, AB prescription patterns, no focus on	2010	The epidemiology of urinary tract infection	B. Foxman	Nature Reviews Urology	Review
No use of ASPT as defined but only UTI management, AB prescription patterns, no focus on ASPT	2010	Presentation, pattern, and natural course of severe symptoms, and role of antibiotics and antibiotic resistance among patients presenting with suspected uncomplicated urinary tract infection in primary care: observational study	P Little, R Merriman, S Turner, K Rumsby, G Warner, J A Lowes, H Smith, C Hawke, G Leydon, M Mullee, M V Moore	BMJ	Original
No use of ASPT as defined but only UTI management, AB prescription patterns, no focus on	2011	Antimicrobial management and appropriateness of treatment of urinary tract infection in general practice in Ireland	A. Vellinga, M. Cormican, B. Hanahoe, K. Bennett and A. W. Murphy	BMC Family Practice	Original
No use of ASPT as defined but only UTI management, AB prescription patterns, no focus on	2012	Clinical practice. Uncomplicated Urinary Tract Infection	T. M. Hooton	NEJM	Review
No use of ASPT as defined but only UTI management, AB prescription patterns, no focus on	2013	Awareness of antibiotic resistance and antibiotic prescribing in UTI treatment: A qualitative study among primary care physicians in Sweden	I. Björkman, J. Berg, N. Viberg, C. Stålsby Lundborg	Scandinavian Journal of Primary Health Care	Original
No use of ASPT as defined but only UTI management, AB prescription patterns, no focus on ASPT	2017	Variations in presentation, management, and patient outcomes of urinary tract infection: a prospective four-country primary care observational cohort study	C. C. Butler, N. Francis, E. Thomas-Jones, C. Llor, E. Bongard, M. Moore, P.Little, J. Bates, M. Lau, T. Pickles, M. Gal, M. Wootton, N. Kirby, D. Gillespie, K. Rumbsy, C. Brugman, K. Hood, T. Verheij	British Journal of General Practice	Original
No use of ASPT as defined but only UTI management, AB prescription patterns, no focus on	2017	Antibiotic resistance rates and physician antibiotic prescription patterns of uncomplicated urinary tract infections in southern Chinese primary care	C. K. M. Wong, K. Kung, P. L. W. Au-Doung, M. Ip, N. Lee, A. Fung, S. Y. S. Wong	Plos one	Original
No use of ASPT as defined but only UTI management, AB prescription patterns, no focus on ASPT	2018	Practice guidelines for the management of adult community-acquired urinary tract infections	F. Caron, T. Galperine, C. Flateau, R. Azria, S. Bonacorsi, F. Bruyère, G. Cariou, E. Clouqueur, R. Cohen, T. Doco-Lecompte, E. Elefant, K. Faure, R. Gauzit, G. Gavazzi, L. Lemaitre, J. Raymond, E. Senneville, A. Sotto, D. Subtil, C. Trivalle, A. Merens, M. Etienne	Med Mal Infect.	Review
No use of ASPT as defined but only UTI management, AB prescription patterns, no focus on ASPT	2018	Effect of 5-Day Nitrofurantoin vs Single-Dose Fosfomycin on Clinical Resolution of Uncomplicated Lower Urinary Tract Infection in Women: A Randomized Clinical Trial.	A. Huttner, A. Kowalczyk, A. Turjeman, T. Babich, C. Brossier, N. Eliakim-Raz, K. Kosiek, B. Martinez de Tejada, X. Roux, S. Shiber, U. Theuretzbacher, E. von Dach, D. Yahav, L. Leibovici, M. Godycki-Cwirko, J. W. Mouton, S. Harbarth	JAMA	Original
No use of ASPT as defined but only UTI management, AB prescription patterns, no focus on ASPT	2018	Is A Combination of Antibiotics and Non-Steroidal Anti-Inflammatory Drugs More Beneficial Than Antibiotic Monotherapy For The Treatment of Female Acute Uncomplicated Cystitis? A Randomized Controlled Pilot Study	K. Ko, W. K. Lee, C. Y. Oh, S. H. Lee, S. T. Cho, W. J. Bang, T. Y. Shin, M. S. Choo, J. S. Cho, Y. G. Lee, D. Y. Yang	Urology journal	Original
No use of ASPT as defined but only UTI management, AB prescription patterns, no focus on	2019	Prescription of antibiotics for urinary tract infection in general practice in Denmark	A. Holm, G- Cordoba & R. Aabenhus	Scandinavian Journal of Primary Health Care	Original
ASPT no specific for UTI	2019	Knowledge, Perceptions and Practices of Community Pharmacists Towards Antimicrobial Stewardship:A Systematic Scoping Review	S- K. Saha, C. Barton, S. Promite, D. Mazza	Antibiotics	Review
ASPT no specific for UTI	2019	General practitioners' accounts of negotiating antibiotic prescribing decisions with patients: a qualitative study on what influences antibiotic prescribing in low, medium and high prescribing practices	M. M. van der Zande, M. Dembinsky, G. Aresi and T. P. van Staa	BMC Family Practice	Original
No use of ASPT as defined but only UTI management, AB prescription patterns, no focus on	2020	Natural history of uncomplicated urinary tract infection without antibiotics: a systematic review	T. Hoffmann, R. Peiris, C. D. Mar, G. Cleo, P. Glasziou	British Journal of General Practice	Review

ASPT no specific for UTI	2020	Barriers and facilitators to the uptake of an antimicrobial stewardship program in primary care: A qualitative study	L. Jeffs, W. McIsaac, M. Zahradnik, A. Senthinathan, L. Dresser, M. McIntyre, D. Tannenbaum, C. Bell, A. Morris, L. Dresser, M. McIntyre, D. Tannenbaum, C. Bell, A. Morris	Plos one	Original
ASPT no specific for UTI	2020	The complex phenomenon of dysrational antibiotics prescribing decisions in German primary healthcare: a qualitative interview study using dual process theory	R. Poss-Doering, M. Kamradt, A. Stuermlinger, K. Glassen, P. Kaufmann-Kolle, E. Andres and M. Wensing	Antimicrobial Resistance and Infection Control	Original
No use of ASPT as defined but only UTI management, AB prescription patterns, no focus on	2020	Uncomplicated urinary tract infection in primary health care: presentation and clinical outcome	H. Kornfält Isberg, K. Hedin, E. Melander, S. Mölstad, A. Beckman	infectious diseases	Original
No use of ASPT as defined but only UTI management, AB prescription patterns, no focus on	2020	Workload, diagnostic work-up and treatment of urinary tract infections in adults during out-of-hours primary care: a retrospective cohort study	M. Spek, J. W. L. Cals, G. J. Oudhuis, P. H. M. Savelkoul, E. G. P. M. de Bont	BMC Family Practice	Original
ASPT no specific for UTI	2022	How can general practitioners reduce antibiotic prescribing in collaboration with their patients?	C. Del Mar, T. Hoffmann, M. Bakhit	AJGP	Review
No use of ASPT as defined but only UTI management, AB prescription patterns, no focus on	2022	Educational Interventions to Reduce Prescription and Dispensing of Antibiotics in Primary Care: A Systematic Review of Economic Impact	V. Rocha, M. Estrela, V. Neto, F. Roque, A. Figueiras and M. T. Herdeiro	Antibiotics	Review
ASPT no specific for UTI	2018	An underutilised resource for Antimicrobial Stewardship: a 'snapshot' of the community pharmacists' role in delayed or 'wait and see' antibiotic prescribing	M. L. Avent, J. Fejzic, M. L. van Driel	International Journal of Pharmacy Practice	Original

Table S9. Data extraction of all sources

Year	Title	Author	Journal	Language	type of study	setting	country	sample size	population	Aims	Intervention / Focus	Outcomes of interest
2010	Symptomatic treatment (ibuprofen) or antibiotics (ciprofloxacin) for uncomplicated urinary tract infection? results of a randomized controlled pilot trial	J. Bleidorn, I. Gagyor, M. M. Kochen, K. Wegscheider and E. Hummers-Pradier	BMC Medicine	English	Randomized controlled trial	GP / PC	Germany	79	women aged 18-85 yrs otherwise healthy not pregnant with UTI symptoms	to prove the equivalence of ibuprofen and ciprofloxacin for uUTI with regard to symptom resolution (pilot)	Ibuprofen (3×400 mg) vs ciprofloxacin (2 x 250mg (+1 placebo) for 3 days	Symptom resolution<br>Burden of symptoms<br>Relapses<br>SAE/AE<br>Secondary antibiotic treatments<br>Re-consultation
2010	Women's views about management and cause of urinary tract infection: qualitative interview study	G. M. Leydon, S. Turner, H. Smith and P. Little	BMJ	English	Qualitative	GP / PC	UK	21 (20 valid interviews)	women aged 21-64 yrs participating in an RCT (delayed prescribing group)	to explore the views of women with UTI on the acceptability of different strategies for managing the infection	delayed AB vs immediate AB	Positive view about delay AB<br>Fear of progression or worsening of symptoms<br>Prior positive experience / satisfaction with AB treatment<br>Alternative to antibiotics are welcomed<br>Avoiding side effects of AB<br>Delayed AB as a security (in case of emergency or when tryng alternatives)<br>Not feeling considered / not taken seriously<br>Feeling that it's too late to delay when consulting
2010	Effectiveness of five different approaches in management of urinary tract infection: randomised controlled trial	P. Little, M. V. Moore, S. Turner, K. Rumsby, G. Warner, J. A. Lowes, H. Smith, C. Hawke, G. Leydon, A. Arscott, D. Turner and M. Mullee	BMJ	English	Randomized controlled trial	GP / PC	UK	309	not pregnant women aged 18-70 yrs with uUTI symptoms randomised to 5 management approaches (Immediate AB, delayed AB, symptom score, dipstick, urine culture)	to assess the impact of five different management strategies on UTI	delayed AB vs immediate AB	Symptom resolution<br>Burden of symptoms<br>AB consumption (total number of courses)<br>Re-consultation
2010	Cost effectiveness of management strategies for urinary tract infections: results from randomised controlled trial	D. Turner, P. Little, J. Raftery, S. Turner, H. Smith, K. Rumsby and M. Mullee	BMJ	English	Randomized controlled trial	GP / PC	UK	309	not pregnant women aged 18-70 yrs with uUTI symptoms randomised to 5 management approaches (Immediate AB, delayed AB, symptom score, dipstick, urine culture)	to assess the cost-effectiveness of different management strategies	delayed AB vs immediate AB	Consultation length<br>Urine samples taken<br>Dipstick test carried out<br>Re-consultation<br>Secondary antibiotic treatments<br>Re-consultation<br>Costs
2011	Update in adult urinary [illegible]	L.E. Nicolle	Current Infectious [illegible]	English	Reviews (Literature, Narrative reviews [illegible]	ambulatory / [illegible]	Canada	n.a.	evidence of UTI [illegible]	to provide a review of [illegible]	ASPT vs Antibiotics	Symptom resolution<br>AB consumption (total number of courses)<br>Costs

2011	tract infection	L.E. Nicolle	Disease Reports	English	reviews, Systematic Reviews, Meta analysis)	outpatient	Canada	n.a.	evidence of UTI	all UTI	ASPT vs Antibiotics	Alternative to antibiotics are welcomed by women but the rationale should be provided<br>Symptom resolution
2013	Women with symptoms of uncomplicated urinary tract infection are often willing to delay antibiotic treatment: a prospective cohort study	B. J. Knottnerus, S. E. Geerlings, E. P. Moll van Charante and G. ter Riet	BMC Family Practice	English	Cohort study (prospective, retrospective)	GP / PC	Netherlands	176	women aged 16-89 yrs otherwise healthy not pregnant contacting the GP for symptoms of UTI lasting no longer than 7 days	to investigate how many women presenting with UTI symptoms were willing to delay antibiotic treatment	delayed AB asked (137 patients) vs. not asked	Willing to delay antibiotics<br>Clinical resolution<br>PN<br>AB consumption (total number of courses)<br>Factors associated with ASPT
2014	Cystitis: antibiotic prescribing, consultation, attitudes and opinions	C. S. Willems, J. van den Broek D'Obrenan, M. E. Numans, T. J. Verheij and A. W. van der Velden	Family Practice	English	Cross-sectional	GP / PC	Netherlands	231	women otherwise healthy with uUTI symptoms	to investigate attitudes and opinions of women with respect to uUTI management and antibiotics	delayed AB vs immediate AB	Willing to delay antibiotics<br>Patients used AB to reduce pain<br>Factors associated with ASPT
2015	Antimicrobial Stewardship in Outpatient Settings: A Systematic Review	D. M. Drekonja, G. A. Filice, N. Greer, A. Olson, R. MacDonald, I. Rutks and T. J. Wilt	Infection control & hospital epidemiology	English	Reviews (Literature, Narrative reviews, Systematic Reviews, Meta analysis)	ambulatory / outpatient	USA	6 studies (UTI)	English language studies evaluating stewardship programs in outpatient settings	to evaluate the effect of outpatient antimicrobial stewardship programs on prescribing, patient, microbial outcomes, and costs	ASPT vs Antibiotics	AB consumption (total number of courses)<br>Re-consultation
2015	Incidence, severity, help seeking, and management of uncomplicated urinary tract infection: a population-based survey	C. C. Butler, M. K. Hawking, A. Quigley and C. A. McNulty	British Journal of General Practice	English	Cross-sectional	population / community	UK	892	women aged ≥16 yrs	to describe incidence and severity, help seeking, and management of UTI	ASPT vs Antibiotics	AB consumption (total number of courses)<br>Factors associated with ASPT<br>Providing information about UTI management and ASPT
2015	Ibuprofen versus fosfomycin for uncomplicated urinary tract infection in women: randomised controlled trial	I. Gagyor, J. Bleidorn, M. M. Kochen, G. Schmiemann, K. Wegscheider and E. Hummers-Pradier	BMJ	English	Randomized controlled trial	GP / PC	Germany	484	women aged 18-65 yrs otherwise healthy not pregnant with UTI symptoms	to assess whether the number of AB issued for UTI can be reduced by symptomatic treatment with ibuprofen and without an increase in symptom burden, recurrences, or complications.	ibuprofen (3×400 mg for 3 days) vs. fosfomycin (1X3g for 1 day)	AB consumption (total number of courses)<br>Burden of symptoms<br>Symptom resolution<br>PN<br>SAE/AE
2015	Delayed/back up antibiotic prescriptions: what do the public think?	C. A. McNulty, D. M. Lecky, M. K. Hawking, A. Quigley and C. C. Butler	BMJ Open	English	Cross-sectional	population / community	UK	1625 (51% female)	aged ≥15 yrs	to describe the general public's understanding, acceptance and use of delayed antibiotics.	delayed AB vs immediate AB	Awareness about ASPT<br>Factors associated with ASPT<br>Willing to delay antibiotics
	Using qualitative insights to change practice:	S. Duane, C. Domegan, A. Callan,							aged 18-70 yrs; if women, non	to explore the culture of antibiotic prescribing		AB resistance as a justification for ASPT<br>GP and patient behaviour / beliefs mismatch

2016	to change practice: exploring the culture of antibiotic prescribing and consumption for urinary tract infections	S. Galvin, M. Cormican, K. Bennett, A. W. Murphy and A. Vellinga	BMJ Open	English	Qualitative	GP / PC	Ireland	15 GP; 42 community members	women, non pregnant, with direct or indirect experience of uUTI) and GPs	and consumption in the community for UTI from the perspective of the GP and community member.	ASPT vs Antibiotics	Prescriptions because of reductions of re-consultations<br>Different types of GPs and patients have different views<br>Experienced patients want AB<br>Private patients are expecting a prescription
2016	Predicting antibiotic prescription after symptomatic treatment for urinary tract infection: development of a model using data from an RCT in general practice	I. Gagyor, J. Haasenritter, J. Bleidorn, W. McIsaac, G. Schmiemann, E. Hummers-Pradier and W. Himmel	British Journal of General Practice	English	Randomized controlled trial	GP / PC	Germany	235	women aged 18-65 yrs otherwise healthy not pregnant with UTI symptoms and without NSAIDs	to investigate whether there are differences between females with a UTI who were subsequently prescribed antibiotics and those who recovered with symptomatic treatment only	Ibuprofen (3x400 mg for 3 days) vs. Antibiotics	Factors associated with secondary AB
2016	Recurrent urinary tract infections and complications after symptomatic versus antibiotic treatment: follow-up of a randomised controlled trial	J. Bleidorn, E. Hummers-Pradier, G. Schmiemann, B. Wiese and I. Gagyor	German Medical Science	English	Cohort study (prospective, retrospective)	GP / PC	Germany	386	women aged 18-65 yrs otherwise healthy not pregnant with UTI symptoms	to assess the influence of initial (non)antibiotic treatment on recurrent UTI rates and pyelonephritis after day 28 up to 6 months after trial participation	ibuprofen (3×400 mg) and no AB in follow-up vs fosfomycin (1x3 g for 3 days) and AB in follow-up	Relapses<br>PN<br>Factors associated with relapses / recurrence
2016	Exploring Experiences of Delayed Prescribing and Symptomatic Treatment for Urinary Tract Infections among General Practitioners and Patients in Ambulatory Care: A Qualitative Study	S. Duane, P. Beatty, A. W. Murphy and A. Vellinga	Antibiotics	English	Qualitative	GP / PC	Ireland	7 GPs; 14 patients	GPs and UTI patients - (otherwise healthy adult women presenting at GP with UTI symptoms) recruited from 1 practice	to explore GP and patient attitudes and experiences regarding the use of delayed or back-up antibiotic and symptomatic treatment for UTI	delayed AB (24-72h) and symptomatic treatment vs. Immediate AB	Treating with AB if UTI is confirmed<br>Delayed prescription to ensure the patient will not re-consult<br>Patients with previous experience of UTI expect AB<br>Delayed in patients with symptoms but negative dipstick<br>Fear of progression or worsening of symptoms<br>Patients feel better with symptomatic treatment and reduce reliance on AB<br>Patients have mixed feelings about delayed prescribing (patients without experiences)<br>Patients are satisfied with AB prescription, as they feel they need<br>Uncertainty with accepting symptomatic treatment<br>Acceptance depending on the severity of symptoms
2017	Symptomatic treatment of uncomplicated lower urinary tract infections in the ambulatory setting: randomised, double blind trial	A. Kronenberg, L. Butikofer, A. Odutayo, K. Muhlemann, B. R. da Costa, M. Battaglia, D. N. Meli, P. Frey, A. Limacher, S. Reichenbach and P. Juni	Bmj	English	Randomized controlled trial	GP / PC	Switzerland	253	not pregnant women aged 18-70 yrs with uUTI symptoms	to investigate whether symptomatic treatment with NSAIDs is non-inferior to antibiotics in the treatment of uUTI	Diclofenac retard (75 mg for 3 days)+Delay antibiotic after 3 days if symptoms persisted vs. Norfloxacin 400 mg X 3 days	Symptom resolution<br>AB consumption (total number of courses)<br>Clinical resolution<br>Re-consultation<br>Patient satisfaction<br>SAE/AE

2017	Misconceptions of Spanish general practitioners' attitudes toward the management of urinary tract infections and asymptomatic bacteriuria: an internet-based questionnaire study	C. Llor, A. Moragas, S. Hernandez, S. Crispi and J. M. Cots	Revista Espanola de Quimioterapia	English	Cross-sectional	GP / PC	Spain	1235	GPs	to analyse the management of uncomplicated and complicated UTIs and asymptomatic bacteriuria	delayed AB vs immediate AB	Delayed and no treatment depends on CFU from culture analysis
2017	Results of a randomized, prospective, doubledummy, double-blind trial to compare efficacy and safety of a herbal combination containing Tropaeoli majoris herba and Armoraciae rusticanae radix with co-trimoxazole in patients with acute and uncomplicated cystitis	R. Stange, B. Schneider, U. Albrecht, V. Mueller, J, Schnitker and A. Michalsen	Research and Reports in Urology	English	Randomized controlled trial	ambulatory / outpatient	Germany	96	aged 18–70 yrs with UTI symptoms (90% female)	to demonstrate non-inferiority of an herbal combination to an antibiotic (co-trimoxazole) in acute UTI	herbal combination (80 mg horseradish root and 200 mg nasturtium herb powder) five tablets, four times per day for 7 days following by 21 days without treatment vs. co-trimoxazole (160 mg trimethoprim & 800 mg sulfamethoxazole) 2 times per 3 days+ placebo	Clinical resolution<br>Symptom resolution<br>Relapses<br>Safety<br>Patient satisfaction
2018	Non-Antibiotic Herbal Therapy (BNO 1045) versus Antibiotic Therapy (Fosfomycin Trometamol) for the Treatment of Acute Lower Uncomplicated Urinary Tract Infections in Women: A Double-Blind, Parallel-Group, Randomized, Multicentre, Non-Inferiority Phase III Trial	F. M. Wagenlehner, D. Abramov-Sommariva, M. Holler, H. Steindl and K. G. Naber	Urologia Internationalis	English	Randomized controlled trial	ambulatory / outpatient	Germany / Poland / Ukraine	588	not pregnant women aged 18-70 yrs with uUTI symptoms, not recurrent	to determine whether herbal therapy with Canephron® N (BNO 1045) is non-inferior to fosfomycin trometamol (FT) in treating acute lower uUTI	Canephron® N (BNO 1045) 2 tablets X 3 for 7 days. vs. Fosfomycin (3 g) 1 day. Concomitant symptomatic treatment permitted was paracetamol.	Secondary antibiotic treatments<br>PN<br>Burden of symptoms<br>Safety<br>Clinical resolution
2018	Ibuprofen versus Pivmecillinam for Uncomplicated Urinary Tract Infection in Women - A Double-Blind, Randomized Non-Inferiority Trial	I. Vik, M. Bollestad, N. Grude, A. Baerheim, E. Damsgaard, T. Neumark, L. Bjerrum, G. Cordoba, I. C. Olsen and M. Lindbaek	PONE	English	Randomized controlled trial	GP / PC	Norway / Sweden / Denmark	359	not pregnant women aged 18-60 yrs with uUTI symptoms	to assess whether treatment with ibuprofen was non-inferior to pivmecillinam in achieving symptomatic resolution by day 4	ibuprofen (3 X 600mg X 3 days) vs. Pivmecillinam (3 days, 1 tablet -200 mg). Paracetamol allowed	Symptom resolution<br>Burden of symptoms<br>Clinical resolution<br>Secondary antibiotic treatments<br>PN
2019	Use of delayed antibiotic prescription in primary care: a cross-sectional study	M. de la Poza Abad, G. Mas Dalmau, I. Gich Saladich, L. Martinez Garcia, C. Llor and P. Alonso-Coello	BMC Family Practice	English	Cross-sectional	GP / PC	Spain	215	health professionals in PC	to identify delay prescribing approach used in PC	delayed AB (45%) vs. immediate AB	Delays prescription reduces inappropriate antibiotic use<br>Delay prescription is a good strategy to optimize the use of available resources<br>Patients are satisfied with delay prescription<br>Prior experience with delay prescription as a factor for more favorable perspective on delay<br>Delay prescription changes patient perceptions of AB

												Physicians use more delay prescription than nurses
2019	Uva-ursi extract and ibuprofen as alternative treatments for uncomplicated urinary tract infection in women (ATAFUTI): a factorial randomized trial	M. Moore, J. Trill, C. Simpson, F. Webley, M. Radford, L. Stanton, T. Maishman, A. Galanopoulou, A. Flower, C. Eyles, M. Willcox, A. D. Hay, E. van der Werf, S. Gibbons, G. Lewith, P. Little and G. Griffiths	Clinical Microbiology & Infection	English	Randomized controlled trial	GP / PC	UK	382	not pregnant women aged 18-70 yrs with uUTI symptoms, not recurrent	to investigate if offering symptomatic therapy alongside a delayed prescription would relieve symptoms and reduce the consumption of AB for adult women presenting with UTI	1) Uva-ursi (total daily dose of 3600mg for 3-5 days) + advice to take ibuprofen (daily dose of 1200 mg); 2) Placebo + advice to take ibuprofen; 3) Uva-ursi + no advice to take ibuprofen; 4) Placebo + no advice to take ibuprofen.<br>+ Delayed prescription	Burden of symptoms<br>Symptom resolution<br>AB consumption (total number of courses)<br>Re-consultation<br>SAE/AE
2020	Guideline of the Swiss Society of Gynaecology and Obstetrics (SSGO) on acute and recurrent urinary tract infections in women, including pregnancy	C. Betschart, W. C. Albrich, S. Brandner, D. Faltin, A. Kuhn, D. Surbek and V. Geissbuehler	Swiss Medical Weekly	English	Guidelines / Best practice / Commentary	ambulatory / outpatient	Switzerland	n.a.	evidence of UTIs and recurrent UTIs in women	to provide answers for every day clinical questions concerning UTIs, especially for obstetricians and gynaecologists.	ASPT vs Antibiotics	Recommend NSAIDs, delay as an option. Discuss with patients. Consider higher risk for PN
2020	Diagnosis and Management of UTI in Primary Care Settings-A Qualitative Study to Inform a Diagnostic Quick Reference Tool for Women Under 65 Years	E. Cooper, L. Jones, A. Joseph, R. Allison, N. Gold, J. Larcombe, P. Moore and C. A. M. McNulty	Antibiotics	English	Qualitative	GP / PC	UK	57	GPs, health care assistants, nurses, nurse prescribers, other (8 focus group)	to explore factors influencing the diagnosis and management of UTIs in primary care	delayed AB vs immediate AB	Patients are unaware that UTI can resolve by its own<br>Patients react differently to no AB approach, compated to RTI as there are more consistent messaging / campaigns<br>Only the patients, in whom self-care didn't work come for a<br>Fear of progression or worsening of symptoms<br>Cranberry juice is effective because hydration is a way to “flush out” the bladder/infection.<br>Delay antibiotics when waiting for results from urine culture if symptoms were mild.<br>Factors that would influence the clinician’s decision to provide an immediate antibiotic: comorbidities / pregnancy; cost of treatment; severity of symptoms; age of patient; time before the weekend.<br>Not receiving an antibiotic immediately is becoming widely more accepted.<br>Delayed/back-up prescription for UTIs is not usual<br>Patients expect to get AB<br>Patients with previous experience of UTI expect AB
2020	Management of urinary tract infections: what do doctors recommend and	I. Gagyor, S. Strube-Plaschke, K.	BMC Infectious	English	Cohort study (prospective	GP / PC	Germany	120	not pregnant women aged ≥18 yrs with uUTI invited to take a	to compare management of uUTI as recommended by	NSAIDs vs. Antibiotics	Women take NSAIDs independent of GP recommendation<br>Women taking NSAIDs had higher symptom score at baseline<br>Faster / immediate symptom relief taking AB<br>Decision to take an AB was independent from later UTI confirmation by culture

2020	patients do? An observational study in German primary care	Rentzsch and W. Himmel	Infectious Diseases	English	(prospective, retrospective)	GP / PC	Germany	[illegible]	invited to take a survey for 7 days and followed-up at 28 days.	the physicians with the patients' management strategies at home.	NSAIDs vs. Antibiotics	no difference in re-occurence of UTI<br>Many women did not follow GP recommendation<br>Dipstick results, urine culture results and symptom severity were not strongly associated with women's decisions for or against the recommended treatment
2020	Optimising management of UTIs in primary care: a qualitative study of patient and GP perspectives to inform the development of an evidence-based, shared decision-making resource	D. M. Lecky, J. Howdle, C. C. Butler and C. A. McNulty	British Journal of General Practice	English	Qualitative	GP / PC	UK	29 women; 20 GP	women aged ≥16 yrs with UTI within 12 months who consulted a GP for the symptoms. GPs with experience in UTI	to develop an evidence-based, shared decision-making intervention leaflet to optimise management of uncomplicated UTI for women <65 yrs in PC	ASPT vs Antibiotics	Patients expect to get AB<br>Late presentation of patients and fear of PN foster AB prescription<br>Patients with previous experience of UTI expect AB<br>Lack of materials and knowledge to improve the communication<br>Time pressure during a consultation favour AB prescription
2020	Predicting the use of antibiotics after initial symptomatic treatment of an uncomplicated urinary tract infection: analyses performed after a randomised controlled trial	I. Vik, I. Mdala, M. Bollestad, G. C. Cordoba, L. Bjerrum, T. Neumark, E. Damsgaard, A. Baerheim, N. Grude and M. Lindbaek	BMJ Open	English	Secondary data analysis	GP / PC	Norway / Sweden / Denmark	143	not pregnant women aged 18-60 yrs with uUTI symptoms, initially treated with ibuprofen	to predict antibiotic use after initial treatment with ibuprofen	Ibuprofen and no AB	Secondary antibiotic treatments<br>Factors associated with secondary AB
2020	Is Non-Steroidal Anti-Inflammatory Therapy Non-Inferior to Antibiotic Therapy in Uncomplicated Urinary Tract Infections: a Systematic Review	M. R. Carey, V. M. Vaughn, J. Mann, W. Townsend, V. Chopra and P. K. Patel	Journal of General Internal Medicine	English	Reviews (Literature, Narrative reviews, Systematic Reviews, Meta analysis)	ambulatory / outpatient	USA	5 studies	RCT comparing antibiotics versus NSAIDs for treatment of uUTIs	to understand whether NSAIDs may reduce overall antibiotic prescribing for uUTI	NSAIDs vs. Antibiotics	PN<br>Symptom resolution<br>AB consumption (total number of courses)
2021	Construction of a patient decision aid for the treatment of uncomplicated urinary tract infection in primary care	Y. M. Vincent, A. Frachon, C. Buffeteau and G. Conort	BMC Family Practice	English	Mixed	GP / PC	France	34 studies, 16-24 women	women	to create a patient decision aid (PtDA) used in primary care settings to make a shared decision between practitioners and women about whether or not to treat uUTI with antibiotics.	ASPT vs Antibiotics<br>NSAIDs vs. Antibiotics<br>Phytotherapy vs. Antibiotics<br>ASPT vs Antibiotics	Symptom resolution<br>PN<br>Burden of symptoms<br>Interest in Alternatives to Antibiotics
											ASPT: 1) delay: urine culture first wait for results for 2 days, then [illegible]	Cost-effectiveness<br>AB consumption (total number of courses)

2021	Role of antibiotic resistance in urinary tract infection management: a cost-effectiveness analysis	R. Wang and C. LaSala	American Journal of Obstetrics & Gynecology	English	Mixed	ambulatory / outpatient	USA	n.a.	case scenario: decision tree model	to evaluate the cost effectiveness of UTI testing and treatment strategies with a focus on antibiotic resistance	treat with antibiotics based on culture results; 2) symptomatic treatment first for 2 days, followed by culture-directed antibiotics, if symptoms persist; AB: 3) empirical treatment; 4) urine culture and empirical treatment	Burden of symptoms
2021	Herbal treatment with uva ursi extract versus fosfomycin in women with uncomplicated urinary tract infection in primary care: a randomized controlled trial	I. Gagyor, E. Hummers, G. Schmiemann, T. Friede, S. Pfeiffer, K. Afshar and J. Bleidorn	Clinical Microbiology & Infection	English	Randomized controlled trial	GP / PC	Germany	398	women aged 18-75 yrs with uUTI symptoms	to explore whether initial treatment with the herbal drug uva-ursi reduces antibiotic use in women with uUTI without increasing symptom burden and complication frequency compared with antibiotic treatment	Uva Ursi (105 mg arbutin, 3X2 tablets daily for 5 days) vs. Fosfomycin powder (3 g) as a single dose or respective placebos.	AB consumption (total number of courses)<br>Burden of symptoms<br>PN<br>SAE/AE<br>Symptom resolution<br>Relapses<br>Re-consultation<br>Patient taking analgetics
2021	Does cranberry extract reduce antibiotic use for symptoms of acute uncomplicated urinary tract infections (CUTI)? A feasibility randomised trial	O. Gbinigie, J. Allen, N. Williams, M. Moore, A. D. Hay, C. Heneghan, A. M. Boylan and C. C. Butler	BMJ Open	English	Randomized controlled trial	GP / PC	UK	46	women aged ≥18 yrs wiith uUTI symptoms	to determine the feasibility of conducting a randomised trial of the effectiveness of cranberry extract in reducing antibiotic use by women with symptoms of acute uUTIs	1) immediate AB (control); 2) Immediate AB + Cranberry (72 mg of proanthocyanidins daily X 7 days); 3) Cranberry + delayed AB	AB consumption (total number of courses)<br>Cranberry could help UTI symptoms<br>Burden of symptoms<br>Symptom resolution<br>SAE/AE
2021	Symptomatic treatment (using NSAIDS) versus antibiotics in uncomplicated lower urinary tract infection: a meta-analysis and systematic review of randomized controlled trials	A. M. C. Ong Lopez, C. J. L. Tan, A. S. Yabon, 2nd and A. N. Masbang	BMC Infectious Diseases	English	Reviews (Literature, Narrative reviews, Systematic Reviews, Meta analysis)	ambulatory / outpatient	n.a.	1165 participants in 4 studies	evidence of uUTI in not pregnant women aged ≥18 yrs	to determine whether NSAIDs can serve as an effective and safe option in the treatment of uncomplicated lower UTI among non-pregnant women compared to antibiotics.	NSAIDs vs. Antibiotics	Symptom resolution<br>PN<br>Clinical resolution<br>Secondary antibiotic treatments
2021	Identifying women's preferences for treatment of urinary tract infection: a discrete choice experiment	H. van der Worp, D. Brandenbarg, P. A. Boek, J. H. W. Braams, L. J. F. Brink, J. Keupers and M. H. Blanker	BMJ Open	English	Mixed	population / community	Netherlands	833	women aged ≥18 yrs	to identify the preferences of women regarding management of UTIs	ASPT vs Antibiotics	Avoiding AMR<br>Symptom resolution<br>Willing to delay antibiotics
	Impact of the COVID-19 pandemic on community [illegible]	A. J. Borek, K. Maitland, M. McLeod, A. Campbell, B. [illegible]								to explore general practicioners views on, and experiences of, the [illegible]		Overall, no impact of the pandemic on UTI treatment with AB<br>Little engagement with antimicrobial stewardship strategies during the pandemics

2021	antibiotic prescribing and stewardship: A qualitative interview study with general practitioners in England	Hayhoe, C. C Butler, L. Morrell, L. S. J. Roope, A. Holmes, A. S. Walker, S. Tonkin-Crine and On Behalf Of The Step-Up Study Team.	Antibiotics	English	Qualitative	GP / PC	UK	24 interviews, 18 GPs	GPs	impact of the COVID-19 pandemic on antibiotic prescribing and antimicrobial stewardship in English General practice	impact of covid-19 pandemic on AB prescribing	Mixed views about the influence of the pandemics on delayed prescriptions
2021	A pragmatic randomized trial of a primary care antimicrobial stewardship intervention in Ontario, Canada	W. McIsaac, S. Kukan, E. Huszti, L. Szadkowski, B. O'Neill, S. Virani, N. Ivers, R. Lall, N. Toor, M. Shah, R. Alvi, A. Bhatt, Y. Nakamachi and A. M. Morris	BMC Family Practice	English	Randomized controlled trial	GP / PC	Canada	54 GPs, 1682 encounters (280 for UTI)	women otherwise healthy aged ≥18 yrs with uUTI	to evaluate the effectiveness of a multi-faceted intervention for reducing antibiotic prescriptions to adults with respiratory and urinary tract infections	Intervention (provider education, clinical decision aids, and audit and feedback of antibiotic prescribing) vs. Control	Delayed prescription for UTI is more likely with the intervention<br>AB consumption (total number of courses)
2021	Development and evaluation of a primary care antimicrobial stewardship program (PC-ASP) in Toronto, Ontario, Canada	W. J. McIsaac, A. Senthinathan, R. Moineddin, Y. Nakamachi, L. Dresser, M. McIntyre, S. Singh, N. De Oliveira, D. Tannenbaum, J. Bloom, C. Lemieux, P. Marr, M. Levy, M. Mitri, S. Walji, S. Kukan and A. M Morris	JAMMI	English	Intervention	GP / PC	Canada	2419 encounters (374 for UTI)	women otherwise healthy aged ≥18 yrs with uUTI	to detail the development of the PC-ASP and an assessment of its effectiveness on antibiotic prescribing for six common infections in adults.	Intervention (multi-faceted educational program) vs. Control	Delayed prescriptions<br>AB consumption (total number of courses)
2021	Uncomplicated urinary tract infection in women	T. C. Hoffmann, M. Bakhit and C. Del Mar	Bmj	English	Guidelines / Best practice / Commentary	GP / PC	Australia	n.a.	evidence of uUTI in not pregnant women	to outline how to identify uUTI in non-pregnant women aged 18-65 yrs and discuss options with women to help them make an informed decision about its management	ASPT vs Antibiotics	Willing to delay antibiotics<br>Symptom resolution
2022	Cross-sectional internet survey exploring women's knowledge, attitudes and practice regarding urinary tract infection-related symptoms in the Netherlands	S. M. L. Cox, M. van Hoof, A. F. K. Lo, G. J. Dinant, G. J. Oudhuis, P. Savelkoul, J. W. L. Cals and E. de Bont	BMJ Open	English	Cross-sectional	GP / PC	Netherlands	975	women aged ≥18 yrs	to explore women's attitudes and experiences regarding UTIs, in order to determine patients' willingness to accept delayed antibiotic prescriptions.	ASPT vs Antibiotics	Willing to delay antibiotics<br>Reason to consult a GP<br>Factors associated with willingness to delay
2022	Efficacy and safety of different therapies of non-steroidal anti-inflammatory drugs against antibiotic monotherapy in the treatment of uncomplicated lower	S. Gautam, R. Shrestha, M. R. Ghani, M. M. Ali, M. Kc, Y. A. Elfert, V. Chong and B. R. Adegbite	SAGE Open Medicine	English	Reviews (Literature, Narrative reviews, Systematic Reviews, Meta	ambulatory / outpatient	n.a.	5 studies	evidence of uUTI in not pregnant women aged ≥18 yrs	to evaluate the efficacy of different NSAIDs therapy (monotherapy or combined with antibiotics) against antibiotics monotherapy to understand the possible role of	NSAIDs vs. Antibiotics	Clinical resolution<br>SAE/AE<br>PN<br>Symptom resolution
											NSAIDs vs. Antibiotics	Clinical resolution<br>SAE/AE<br>PN<br>Symptom resolution
												Clinical resolution

	uncomplicated lower urinary tract infection: A systematic review	Adegbite			analysis)					NSAIDs in managing uUTI and reduce overall antibiotic prescription	NSAIDs vs. Antibiotics	SAE/AE
												PN
												Symptom resolution
											NSAIDs vs. Antibiotics	Clinical resolution
												SAE/AE
2022	Non-antibiotic treatment of acute urinary tract infection in primary care: a qualitative study	O. A. Gbinigie, S. Tonkin-Crine, C. C. Butler, C. J. Heneghan and A. M. Boylan	British Journal of General Practice	English	Qualitative	GP / PC	UK	26	women aged ≥18 yrs with uUTI in the preceding 12 months	to establish women's thoughts on and experience of non-antibiotic treatment for uUTIs.	ASPT vs Antibiotics	Wanting a cure for the UTI
												Conflict between cure and be effective and avoiding antibiotics
												OTC are more 'natural' and easily accessible than AB, but less reliably effective
												For mild symptoms drinking fluids or eating cranberrys is enough
												Not linking analgesia with treating a UTI
												Patients with previous experience of UTI expect AB
												Healthcare professionals tended to express negative or neutral sentiments towards ASPT
												Fear of progression or worsening of symptoms
												First trying alternatives and then if not working ask for antibiotics
												Concern about the taste of cranberry juice, and particularly the sugar content
												Delayed AB as a security (in case of emergency or when tryng alternatives)
												A shorter delay (2-3) is preferred than a longer one
												Views of non-AB are influenced by the views and recommendations of the healthcare practitioner
2022	Reducing antibiotic use in uncomplicated urinary tract infections in adult women: a systematic review and individual participant data meta-analysis	Y. Kausner, C. Rover, J. Heinz, E. Hummers, T. P. A. Debray, A. D. Hay, S. Heytens, I. Vik, P. Little, M. Moore, B. Stuart, F. Wagenlehner, A. Kronenberg, S. Ferry, T. Monsen, M. Lindbaek, T. Friede and I. Gagyor	Clinical Microbiology & Infection	English	Reviews (Literature, Narrative reviews, Systematic Reviews, Meta analysis)	GP / PC	Germany	3602 (8 studies)	RCTs investigating any strategies to reduce antibiotics	to estimate the effect of these (analgesics, herbal formulations, delayed prescription of antibiotics, and placebo) strategies and to identify symptoms, signs, or other factors that indicate a benefit from these strategies	ASPT vs Antibiotics	Clinical resolution
												Symptom resolution
												Secondary antibiotic treatments
												PN
												Relapses
												AB consumption (total number of courses)
												SAE/AE
												Burden of symptoms
2022	Medical Treatment for Urinary Tract Infections	J. Ross and D. Hickling	Urologic Clinics of North America	English	Guidelines / Best practice / Commentary	ambulatory / outpatient	Canada	n.a.	evidence of UTI	to provide a review of pharmacologic treatment and prevention of UTI with both antimicrobial and nonantimicrobial-based therapies	NSAIDs vs. Antibiotics	Symptom resolution
												PN
												AB consumption (total number of courses)
	Why d-Mannose May Be											Symptom resolution

2022	as Efficient as Antibiotics in the Treatment of Acute Uncomplicated Lower Urinary Tract Infections-Preliminary Considerations and Conclusions from a Non-Interventional Study	F. Wagenlehner, H. Lorenz, O. Ewald and P. Gerke	Antibiotics	English	Secondary data analysis	ambulatory / outpatient	Germany	36	women with uUTI	to compare the cure rate of D-mannose monotherapy with that of antibiotics	D-mannose monotherapy vs. D-mannose in combination with antibiotics vs. The combination of D-mannose with other therapeutic measures	Burden of symptoms, Clinical resolution
2022	Experiences of urinary tract infection:A systematic review and meta ethnography	M. Izett-Kay, K. L. Barker, A. McNiven and F. Toye	Neurourol Urodyn	English	Reviews (Literature, Narrative reviews, Systematic Reviews, Meta analysis)	ambulatory / outpatient	UK	16 studies, 1038 participants	evidence of patients aged 13-97 years, men and women	to understand the experience of urinary tract infection (UTI) by synthesizing primary qualitative research findings and developing a conceptual model that illustrates this experience		Avoiding side effects of AB
												Delayed AB as a security (in case of emergency or when tryng alternatives)
												Fear of progression or worsening of symptoms
												AB side effects and resistance
												There is no substitute for AB
												Alternative to antibiotics are welcomed
2022	The Clinical Trial Outcomes of Cranberry, D-Mannose and NSAIDs in the Prevention or Management of Uncomplicated Urinary Tract Infections in Women: A Systematic Review	J. Konesan, L. Liu and K. J. Mansfield	Pathogens	English	Reviews (Literature, Narrative reviews, Systematic Reviews, Meta analysis)	ambulatory / outpatient	Australia	21 studies	evidence of uUTI in women aged ≥18 yrs	to identify, summarise and evaluate the evidence for the outcomes of different nonantibiotic options including cranberry, D-mannose and NSAIDs	ASPT vs Antibiotics	Burden of symptoms
											NSAIDs vs. Antibiotics	Relapses
												Burden of Symptoms
												SAE/AE
											NSAIDs vs. Antibiotics	Burden of Symptoms
												SAE/AE
											NSAIDs vs. Antibiotics	Relapses
												Symptom resolution
2022	Shared decision making for women with uncomplicated Cystitis in Primary Care in the Netherlands: a qualitative interview study	T. M. Z. X. K. van Horrik, B. J. Laan, R. van Seben, G. Rodenburg, E. J. Heeregrave and S. E. Geerlings	BMC Prim Care	English	Qualitative	GP / PC	Netherlands	23 women, 12 GP, 12 assistants	women aged 25-79 yrs otherwise healthy not pregnant with a history of uUTI	to investigate the current management and the use of shared decision management for uUTI in primary care	ASPT vs Antibiotics	Lack of comunication about treatment options (SDM)
												Faster / immediate symptom relief taking AB
												Awareness about ASPT
												Logistical reasons, lack of time, avoiding second visit lead to AB prescription
												Patients wish for AB
												High burden of symptoms lead to AB
												Only the patients, in whom self-care didn't work come for a
												Wish to be (more) involved in the decision making
												Routine to give antibiotics
												Prior positive experience / satisfaction with AB treatment
												Fear of progression or worsening of symptoms
2023	Antibiotic management of urinary tract infections in the post-antibiotic era: a narrative review highlighting diagnostic and antimicrobial stewardship	I. J. Abbott, T. N. Peel, K. A. Cairns and A. J. Stewardson	Clinical Microbiology & Infection	English	Reviews (Literature, Narrative reviews, Systematic Reviews, Meta analysis)	ambulatory / outpatient	Australia	n.a.	evidence of uUTI in women aged ≥18 yrs	to highlight the key clinical decisions involved in the diagnosis and treatment of UTIs in adult women, focusing on clinical effectiveness and both diagnostic and antimicrobial stewardship	ASPT vs Antibiotics	Burden of symptoms
												Patient preferences
												Avoiding AMR
												Symptom resolution

2023	Hospital admission after primary care consultation for community-onset lower urinary tract infection: a cohort study of risks and predictors using linked data	A. Aryee, P. Rockenschaub, J. Robson, M. Priebe, Z. Ahmed, C. N. Fhogartaigh, D. Ball, A. Hayward and L. Shallcross	British Journal of General Practice	English	Cohort study (prospective, retrospective)	GP / PC	UK	169 524 UTI episodes (78% female) in 86'561 patients	patients aged ≥16 yrs	to obtain an accurate estimate of the risk of adverse outcomes following lower UTI in primary care in order to identify patients for whom antibiotic treatment could be safely delayed or avoided as a means of antibiotic stewardship.	delayed AB vs immediate AB	Factors associated with hospital admisson after UTI
2023	Patients' Experiences, Expectations, Motivations, and Perspectives around Urinary Tract Infection Care in General Practice: A Qualitative Interview Study	S. Cox, M. Vleeming, W. Giorgi, G. J. Dinant, J. Cals and E. de Bont	Antibiotics	English	Qualitative	GP / PC	Netherlands	14	women aged 23-78 yrs with UTI	to investigate the experiences, expectations, motivations, and perspectives of patients with UTIs in general practice	ASPT vs Antibiotics	Patients with previous experience of UTI expect AB<br>Lack of knowledge about side-effects of AB<br>Not linking analgesia with treating a UTI<br>Wish to be (more) involved in the decision making<br>Patients wish for AB<br>Lack of knowledge about how harmless UTIs are
2023	Treating urinary tract infections in the era of antibiotic resistance	N. Frimodt-Moller and L. Bjerrum	Expert Review of Antiinfective Therapy	English	Reviews (Literature, Narrative reviews, Systematic Reviews, Meta analysis)	GP / PC	Denmark	n.a.	evidence of women with UTI	to focus on how one can reduce or avoid the use of antibiotics for treatment of suspected UTIs in primary care	NSAIDs vs. Antibiotics	Burden of symptoms<br>PN<br>Symptom resolution<br>AB consumption (total number of courses)
2023	Pharmacists' perspectives on recommending herbal medicines for acute infections: qualitative interview study	X. Y. Hu, M. Logue, E. Maund, M. Santer, M. L. Willcox, S. Islam, T. Stokes and M. Moore	Bjgp Open	English	Qualitative	pharma	UK	18	community pharmacists	to explore community pharmacists' perceptions and experiences of advising patients on management of acute respiratory tract infections and UTIs, and to explore issues regarding use of over-the-counter (OTC) medicines, including herbal medicines	Phytotherapy vs. Antibiotics	Patients expect to get AB<br>Herbal medicine for less severe symptoms<br>Herbal medicine not recommended in high burden of symptoms<br>Herbal medicine not recommended for older patients<br>Evidence of herbal extracts is scarce / inadequate<br>Promoting certain products for profit reasons
2023	Young Women's Attitudes and Behaviors in Treatment and Prevention of UTIs: Are Biomedical Students at an Advantage?	I. Jerkovic, J. Bukic, D. Leskur, A. Seselja Perisin, D. Rusic, J. Bozic, T. Zuvela, S. Vuko, J. Vukovic and D. Modun	Antibiotics	English	Cross-sectional	ambulatory / outpatient	Croatia	392	female students of biomedical school / non-biomedical school	to investigate whether students who study biomedical fields (i.e., medicine, pharmacy science) differ from those whose studies are not connected to the biomedical field. (in relation to UTIs)	biomedical students vs. Non-biomedical students	No need for antibiotics for every UTI<br>Taking self-medication (painkiller, increase liquid intake) prior to consulting a GP<br>It is safe to delay the use of antibiotics and start antibiotic treatment 3 days after the onset of symptoms
	Impact of sociodemographic status and UTI symptoms on								[illegible]	to explore how		

2023	[illegible] women's health-care seeking and management in England: findings from an e-survey conducted during the first year of the COVID-19 pandemic	E. Cooper, B. Read, L. Sanyaolu, H. Ahmed and D. Lecky	Bjgp Open	English	Cross-sectional	ambulatory / outpatient	UK	1096	women aged ≥16 yrs with UTI symptoms in previous year	women's background, symptoms, and severity of symptoms influence UTI reporting and management	delayed AB (10% of women) vs immediate AB (90%)	Factors associated with AB
2023	Management of uncomplicated urinary tract infection in the post-antibiotic era: select non-antibiotic approaches	S. McCallin, T. M. Kessler and L. Leitner	Clinical Microbiology & Infection	English	Reviews (Literature, Narrative reviews, Systematic Reviews, Meta analysis)	ambulatory / outpatient	Switzerland	n.a.	clinical trials published in English language about non-antibiotic treatments for UTI	to highlight non-antibiotic therapies for the treatment of uUTI and relevant indications (prevention and complicated UTI) from recent literature	Phytotherapy vs. Antibiotics	Burden of symptoms<br>AB consumption (total number of courses)<br>PN
											ASPT vs Antibiotics	PN<br>AB consumption (total number of courses)
2023	Management of urinary tract infections in the era of antimicrobial resistance	R. Pothoven	Drug Target Insights	English	Reviews (Literature, Narrative reviews, Systematic Reviews, Meta analysis)	ambulatory / outpatient	Netherlands	n.a.	evidence based material	to summarize evidence-based scientific material, recommendations from the current medical literature, and the latest clinical guidelines on antibiotic and antibiotic-sparing strategies for managing urological infections, including practical approaches to improve the management of patients with acute and recurrent UTIs in routine clinical practice.	ASPT vs Antibiotics	AB consumption (total number of courses)<br>Symptom resolution<br>SAE/AE
2024	General practitioners' decision making managing uncomplicated urinary tract infections in women: a qualitative study	P. K. Kurotschka, J. Hemkeppler, D. Gierszewski, L. Ghirotto and I. Gagyor	Bjgp Open	English	Qualitative	GP / PC	Germany	22	GPs	to explore the decision-making of GPs when managing uUTIs in women	ASPT vs Antibiotics	wait-and- see' approach or no AB treatments if symptoms not severe and no risk for complications<br>Patients expect to get AB<br>Faster / immediate symptom relief taking AB<br>Time pressure during a consultation favour AB prescription<br>Until the middle of the week is more likely to opt for ASPT<br>Reimbursement by statutory health insurance: patients care about the cost of alternatives<br>SDM as a facilitator to discuss options<br>long-term relationships with patients increase acceptance of treatment<br>in-depth consultations as a facilitator for explanation and use of ASPT
											ASPT vs Antibiotics	Burden of symptoms<br>Willing to delay antibiotics

2024	Delayed and Non-Antibiotic Therapy for Urinary Tract Infections: A Literature Review	J. S. Midby and A. R. Miesner	Journal of Pharmacy Practice	English	Reviews (Literature, Narrative reviews, Systematic Reviews, Meta analysis)	GP / PC	USA	13 studies	evidence of uUTI in not pregnant women	to review the literature on delayed antibiotics and non-antibiotic treatments as alternatives to immediate antibiotic prescriptions for uUTI		Health care providers are not aware about ASPT
											delayed AB vs immediate AB	AB consumption (total number of courses)<br>Burden of symptoms<br>Symptom resolution<br>PN
											NSAIDs vs. Antibiotics	Symptom resolution<br>AB consumption (total number of courses)<br>PN
2024	Acute Uncomplicated UTIs in Adults: Rapid Evidence Review	P. K. Kurotschka, I. Gágyor and M.H. Ebell	Am Fam Physician	English	Reviews (Literature, Narrative reviews, Systematic Reviews, Meta analysis)	ambulatory / outpatient	Germany	n.a.	evidence of uUTI in women aged 18-64 yrs	to provide a review of the best available patient-oriented evidence for uUTI	delayed AB vs immediate AB	ASPT are suggested
2020	Revisiting patient expectations and experiences of antibiotics in an era of antimicrobial resistance: qualitative study	O. Boiko, M. Gulliford and C. Burgess	Health Expectations	English	Qualitative	GP / PC	UK	31	patients who consulted general practice for infections: 24 female, 7 male. Age 20-99y, 11 consulted for UTI	to examine patient expectations and experiences of antibiotic prescribing in England.	ASPT vs Antibiotics	Doctors prescribe AB because of the symptoms without considering expectations<br>Mixed views about AB: wish for AB and just seek advice to get better<br>Experienced patients want AB<br>Experience of AB resistance and side effects
2024	Effectiveness of a Combination of Nasturtium Herb and Horseradish Root (Angocin® Anti-Infekt N) Compared to Antibiotics in Managing Acute and Recurrent Urinary Tract Infections: A Retrospective Real-world Cohort Study	N. Kassner, M. Wonnemann, Y. Ziegler, W. Vahlensieck, J. Kranz and K. Kostev	Antibiotics	English	Cohort study (prospective, retrospective)	GP / PC	Germany	13662	patients (83.9% female) with at least one of the following diagnoses: Acute cystitis, other cystitis, Cystitis, unspecified, Urinary tract infection, site not specified	to evaluate whether Angocin® Anti-Infekt N, compared to standard AB treatment after the diagnosis of a UTI, is negatively associated with an early, sporadic, or recurrent UTI, subsequent AB prescriptions, pyelonephritis, or UTI-associated sick leave	Angocin® nasturtium herb (Tropaeoli majoris herba) and horseradish root (2277 patients) vs. Antibiotics (11385 patients)	Relapses<br>Secondary antibiotic treatments<br>Burden of symptoms<br>PN
2022	Clinical experience with a medical device containing xyloglucan, hibiscus, and propolis for the control of acute uncomplicated urinary tract infection-like symptoms	P. Ortega, E. Benito and F. Berrocal	Uro	English	Cohort study (prospective, retrospective)	GP / PC	Switzerland	103	women with a median age of 47years (19-87) attending a primary care physician for a symptomatic episode or recurrence of acute uncomplicated cystitis	to evaluate the efficacy of an oral medical device containing xyloglucan, hibiscus, and propolis in clinical practice with a cohort of women from Switzerland with UTI-like symptoms and the administration of concomitant drugs	Utipro®Plus (xyloglucan 100 mg, gelatin 50 mg, propolis 100 mg, Hibiscus sabdariffa 100 mg; it also contains silicon dioxide, magnesium stearate, and corn starch) 2 X day (1 every 12 h) for 5 days vs concomitant AB and/or NSAIDs (41%)	Re-consultation<br>SAE/AE
	The general practitioner perspective of a multimodal intervention [illegible]	I. Petruschke, K. [illegible]	Zeitschrift fur Evidenz Fortbildung [illegible]						GP with a mean [illegible]	to understand general practitioners [illegible]		Faster / immediate symptom relief taking AB<br>Patients wish for AB

2022	for the adequate use of antibiotics in urinary tract infection - a qualitative interview study	Stichling, A. Greser, I. Gagyor and J. Bleidorn	[illegible] und Qualitat im Gesundheitswesen	German	Qualitative	GP / PC	Germany	10	age of 45 years. 40% were female	perspective of a multimodal intervention for the adequateuse of antibiotics in UTI	ASPT vs Antibiotics	AB prescribed in case of long duration of symptoms
2024	Qualitative study on shared decision making in cystitis management in general practice	T. M. Z. X. K. van Horrik, A. Colliers, M. H. Blanker, E. G. de Bont, A. A. van Driel, B. J. Laan, S. E. Geerlings, R. P. Venekamp, S. Anthierens and T. N. Platteel	Bjgp Open	English	Qualitative	GP / PC	Netherlands	10 GPs, 7 GP assistants, and 15 patients	GPs, GPs assistants, healthy non-pregnant women with a recent history of cystitis (recurrent UTI included)	to identify barriers and facilitators for applying shared decision management in cystitis management in general practice	delayed AB vs immediate AB	Patients expect to get AB
												Non-AB treatments are inferior to AB
												Being unaware of the various non-AB options for cystitis
												SDM is time consuming for GPs and patients
												AB or a wait-and-see policy without involving the patient
												A delayed AB has no added value
												Delayed AB are convenient before weekends and holidays
												Wish to be (more) involved in the decision making
												First trying alternatives and then if not working ask for antibiotics
												Favouring non-pharmacological options for uUTI
												Not being informed about the potential harms or side effects of antibiotics, or about alternative treatment options
												There is no substitute for AB
												Wish to be informed about the likelihood of developing complications, particularly pyelonephritis
												Patients appreciated being involved in treatment decisions
												Delayed AB as a security (in case of emergency or when tryng alternatives)
												Burden of symptoms

2014	Effectiveness of an Association of a Cranberry Dry Extract, D-mannose, and the Two Microorganisms Lactobacillus plantarum LP01 and Lactobacillus paracasei LPC09 in Women Affected by Cystitis<br>A Pilot Study	F. Vicariotto	J Clin Gastroenterol	English	Cohort study (prospective, retrospective)	ambulatory / outpatient	Italy	33	premenopausal women aged > 18 with acute uUTI	to assess the effectiveness of an association of a cranberry dry extract, D-mannose, a gelling complex composed of the exopolysaccharides produced by Streptococcus thermophilus ST10 (DSM 25246) and tara gum, as well as the 2 microorganisms Lactobacillus plantarum LP01 (LMG P-21021) and Lactobacillus paracaseiLPC09 (DSM 24243) in women affected by acute uncomplicated cystitis.	-<br>250 mg D mannose; 500 mg of a high PACs cranberry; 2.5 billion live cells of L. plantarum LP01; 1 billion viable cells each of L. paracasei LPC09 and S. thermophilus ST10 , a strain able to synthesize EPS in the gut lumen, and 250 mg of tara gum 2 doses/day during the first 30 days (acute treatment), and then to continue with 1 sachet per day until the 60th day (long-term treatment) - **antibiotic use was not allowed**	Clinical resolution
2021	Treatment of Urinary Tract Infections with Canephron® in Germany: A Retrospective Database Analysis.	M. Höller, H. Steindl, D. Abramov-Sommariva, F. Wagenlehner, K. G. Naber and K. Kostev	Antibiotics	English	Cohort study (prospective, retrospective)	ambulatory / outpatient	Germany	2320 Canephron® patients and 158,592 antibiotic patients	patients with at least one diagnosis of acute cystitis or UTI (men included)	to evaluate treatment with Canephron® compared to standard antibiotic treatment after diagnosis of acute cystitis or urinary tract infection (UTI)	Canephron® N or UNO (centaury herbs (Centaurium erythraea Rafn, herba), lovage roots (Levisticum officinale Koch, radix), and rosemary leaves (Rosmarinus officinalis Linné, folium) vs.AB - 4% of patients in the Canephron® cohort were also prescribed an analgesic, while in the antibiotic cohort, ~3% received analgesics and just 1% received prescriptions for other drugs (e.g., mannose, methionine, or arbutin-containing drugs)).	Relapses<br>Burden of symptoms<br>AB consumption (total number of courses)<br>PN
2019	Antibiotic-sparing agents for uncomplicated cystitis: uva-ursi and ibuprofen not ready for primetime	R. Datta and M. Juthani-Mehta	Clinical Microbiology & Infection	English	Guidelines / Best practice / Commentary	ambulatory / outpatient	USA	n.a.	evidence of ASPT for uUTI	commentary about antibiotic-sparing agents for uncomplicated cystitis	ASPT vs Antibiotics	Burden of symptoms<br>AB consumption (total number of courses)<br>PN
2024	Therapeutic strategies for uncomplicated cystitis in women	K. G. Naber, J. F. Alidjanov, R. Funfstuck, W. L. Strohmaier, J. Kranz, T. Cai, A. Pilatz and F. M. Wagenlehner	GMS Infectious Diseases	English	Reviews (Literature, Narrative reviews, Systematic Reviews, Meta analysis)	ambulatory / outpatient	Germany	n.a.	evidence of uUTI	to review the literature on the treatment for uUTI (considering also recurrent episodes)	ASPT vs Antibiotics	AB consumption (total number of courses)<br>PN<br>Symptom resolution
2024	The management and treatment of cystitis: same challenges, new strategies	G. Andreoli and A. Huttner	Revue Medicale Suisse	French	Guidelines / Best practice / Commentary	ambulatory / outpatient	Switzerland	n.a.	evidence of uUTI	to discuss challenges and strategies of management and treatment of cystitis	ASPT vs Antibiotics	Uva-ursi cannot be proposed as an alternative to antibiotics<br>Doubt that Canephron was truly noninferior<br>NSAIDs are inferior to AB<br>AB consumption (total number of

												courses)
2020	Management of uncomplicated urinary tract infections with special focus on phytotherapy	K. Béla and M. Attila	Magyar Nőorvosok Lapja	Hungarian	Guidelines / Best practice / Commentary	ambulatory / outpatient	Hungary	n.a.	evidence of uUTI	to review the literature on the treatment for uUTI in women with focus on phytotherapy	Phytotherapy vs. Antibiotics	Symptom resolution
2021	Urinary tract infections - Are antibiotics always necessary?	J. Bleidorn, I. Gagyor and G. Schmiemann	Nieren- und Hochdruckkrankheiten	German	Reviews (Literature, Narrative reviews, Systematic Reviews, Meta analysis)	GP / PC	Germany	n.a.	evidence of uUTI	to provide an overview of the main study results on the non-antibiotic treatment of acute uncomplicated UTI	ASPT vs Antibiotics	Favouring non-pharmacological options for uUTI<br>AB consumption (total number of courses)<br>Non-AB treatments are inferior to AB
2024	Rational therapeutic strategies for urinary tract infections	R. Fünfstück, K. G. Naber, W. L. Strohmaier, W. Pfister and J. Kranz	Nieren- und Hochdruckkrankheiten	German	Reviews (Literature, Narrative reviews, Systematic Reviews, Meta analysis)	ambulatory / outpatient	Germany	n.a.	evidence of UTI	to present recommendations for improved patient care in everyday clinical practice on the basis of important clinical studies and the newly revised guidelines for the diagnosis and treatment of UTIs	ASPT vs Antibiotics	AB consumption (total number of courses)<br>Non-AB treatments are inferior to AB<br>Symptom resolution<br>ASPT are suggested
2023	Urinary tract infection	E. Glover and N. Sheerin	Medicine	English	Reviews (Literature, Narrative reviews, Systematic Reviews, Meta analysis)	ambulatory / outpatient	UK	n.a.	evidence of UTI	to provide a review on recommendations for UTI in UK	ASPT vs Antibiotics	ASPT are suggested
2022	Guidelines for Acute Urinary Tract Infections	A. Gombash and M. Pergola	Current Emergency and Hospital Medicine Reports	English	Guidelines / Best practice / Commentary	ambulatory / outpatient	USA	n.a.	evidence of UTI	to review best practices and provides treatment options	ASPT vs Antibiotics	Burden of symptoms
2022	A Baker's Dozen of Top Antimicrobial Stewardship Intervention Publications in Non–Hospital Care Settings in 2021	S. Green, A. Marx and E. Chahine	Open Forum Infectious Diseases	English	Reviews (Literature, Narrative reviews, Systematic Reviews, Meta analysis)	ambulatory / outpatient	USA	n.a.	evidence of Antimicrobial Stewardship Interventions in non-hospital care setting	to highlight selected high-quality antibiotic stewardship interventions specific to non–hospital care settings	Intervention (provider education, clinical decision aids, and audit and feedback of antibiotic prescribing) vs. Control	Delayed prescription for UTI is more likely with the intervention
2024	Antibiotic Stewardship Interventions for Urinary Tract Infections in Outpatient Settings: A Narrative Review	L. Grigoryan and B. W. Trautner	Infectious Disease Clinics of North America	English	Reviews (Literature, Narrative reviews, Systematic Reviews, Meta analysis)	ambulatory / outpatient	USA	n.a.	evidence of Antimicrobial Stewardship Interventions in non-hospital care setting	to summarize publications and evidence for UTI-focused antibiotic stewardship interventions in the EDs and primary care.	Intervention (provider education, clinical decision aids, and audit and feedback of antibiotic prescribing) vs. Control	Delayed prescription for UTI is more likely with the intervention
2023	Management of Acute Cystitis in the Era of COVID-19	D. Hernández-Hernández and Y. Ortega-González	Current Bladder Dysfunction Reports	English	Reviews (Literature, Narrative reviews, Systematic Reviews, Meta analysis)	ambulatory / outpatient	Spain	n.a.	evidence of UTI	to provide up-to-date information about treatment and follow-up in patients with symptoms suggesting lower urinary tract infection	ASPT vs Antibiotics	PN<br>Symptom resolution<br>Clinical resolution<br>Evidence of herbal extracts is scarce / inadequate

2013	Diagnosis and course of cystitis in healthy non pregnant women	B. Knottnerus, E. M. Van Charante, S. Geerlings and G. Ter Riet	Huisarts en Wetenschap	Dutch	Guidelines / Best practice / Commentary	ambulatory / outpatient	Netherlands	n.a.	evidence of uUTI	to review diagnosis and course of cystitis in healthy non-pregnant women	ASPT vs Antibiotics	AB consumption (total number of courses)<br>ASPT are suggested
2024	European Association of Urology Guidelines on Urological Infections: Summary of the 2024 Guidelines	J. Kranz, R. Bartoletti, F. Bruyere, T. Cai, S. Geerlings, B. Koves, S. Schubert, A. Pilatz, R. Veeratterapillay, F.M.E. Wagenlehner, K. Bausch, W. Devlies, J. Horváth, L. Leitner, G. Mantica, T. Mezei, E. J. Smith and G. Bonkat	European Urology	English	Guidelines / Best practice / Commentary	ambulatory / outpatient	Germany	n.a.	evidence of UTI	guidelines panel to provide evidence-based guidance on the diagnosis, treatment, and prevention of UTI	ASPT vs Antibiotics	ASPT are suggested
2018	Treatment of urinary tract infections: Current recommendations on the use of antibiotics	J. Kranz, K. G. Naber and F. M. E. Wagenlehner	Journal fur Urologie und Urogynakologie	German	Guidelines / Best practice / Commentary	ambulatory / outpatient	Germany	n.a.	evidence of UTI	to review recommendations on the use of antibiotics for UTI	ASPT vs Antibiotics	Secondary antibiotic treatments
2017	Uncomplicated Bacterial Community-acquired Urinary Tract Infection in Adults: Epidemiology, Diagnosis, Treatment, and Prevention	J. Kranz, S. Schmidt and C. Lebert	Dtsch Arztebl Int	English	Guidelines / Best practice / Commentary	ambulatory / outpatient	Germany	n.a.	evidence of uUTI	to review recommendations for uUTI	ASPT vs Antibiotics	Secondary antibiotic treatments<br>ASPT are suggested<br>Burden of symptoms
2017	Epidemiology, diagnostics, therapy, prevention and management of uncomplicated bacterial outpatient acquired urinary tract infections in adult patients: Update 2017 of the interdisciplinary AWMF S3 guideline	J. Kranz, S. Schmidt, C. Lebert, G. Schmiemann and F. M. E. Wagenlehner	Gynakologe	German	Guidelines / Best practice / Commentary	ambulatory / outpatient	Germany	n.a.	evidence of uUTI	to review recommendations on the use of antibiotics for uUTI	ASPT vs Antibiotics	Secondary antibiotic treatments
2017	Epidemiology, diagnostics, therapy, prevention and management of uncomplicated bacterial outpatient acquired urinary tract infections in adult patients : Update 2017 of the interdisciplinary AWMF S3 guideline	J. Kranz, S. Schmidt, C. Lebert, L. Schneidewind, W. Vahlensieck, U. Sester, R. Fünfstück, S. Helbig, W. Hofmann, E. Hummers, M. Kunze, E. Kniehl, K. Naber, F. Mandraka, B. Mündner-Hensen, G. Schmiemann and F. M. E. Wagenlehner	Urologe (Ausg A)	German	Guidelines / Best practice / Commentary	ambulatory / outpatient	Germany	n.a.	evidence of uUTI	to review recommendations on the use of antibiotics for uUTI	ASPT vs Antibiotics	Secondary antibiotic treatments<br>Burden of symptoms<br>ASPT are suggested
					Guidelines / Best					to review definition of		ASPT are suggested

2020	Urinary tract infections	U. Kunter	Nephrologe	German	Guidelines / Best practice / Commentary	ambulatory / outpatient	Germany	n.a.	evidence of UTI	to review definition of UTI, diagnostics and treatment	ASPT vs Antibiotics	ASPT are suggested<br>PN
2014	Are there alternatives to antimicrobial therapy and prophylaxis of uncomplicated urinary tract infections?	K. G. Naber and J. F. Alidjanov	Urologiia (Moscow, Russia).	Russian	Reviews (Literature, Narrative reviews, Systematic Reviews, Meta analysis)	ambulatory / outpatient	Germany	n.a.	evidence of uUTI	to review alternatives to antimicrobial therapy and prophylaxis of uncomplicated urinary tract infections	ASPT vs Antibiotics	Symptom resolution
2019	UTI− quo vadis? New alternatives to treat uncomplicated urinary tract infections	K. G. Naber, Z. Tandogdu, B. Köves and G. Bonkat	Clin Phytoscience	English	Reviews (Literature, Narrative reviews, Systematic Reviews, Meta analysis)	ambulatory / outpatient	Germany	n.a.	evidence of uUTI	evaluating the current scenario and also throwing light on the paradigm shift in the treatment of acute, uncomplicated lower urinary tract infections (uUTI).	ASPT vs Antibiotics	Willing to delay antibiotics<br>NSAIDs are inferior to AB<br>AB consumption (total number of courses)<br>Symptom resolution
2020	Recent research in urological infections	K, G, Naber and F.M. E. Wagenlehner	Nature Reviews Urology	English	Reviews (Literature, Narrative reviews, Systematic Reviews, Meta analysis)	ambulatory / outpatient	Germany	n.a.	evidence of UTI	to review research in urological infections	ASPT vs Antibiotics	Secondary antibiotic treatments<br>Symptom resolution
2014	New aspects on diagnostics and therapy of uncomplicated cystitis	K. G. Naber	Urologe (Ausg A)	German	Guidelines / Best practice / Commentary	ambulatory / outpatient	Germany	n.a.	evidence of uUTI	to review aspects on diagnostics and therapy of uncomplicated cystitis	ASPT vs Antibiotics	Symptom resolution
2022	Systematic review of the effect of D mannose with or without other drugs in the treatment of symptoms of urinary tract infections/cystitis	F. Parazzini, E. Ricci, F. Fedele, F. Chiaffarino, G. Esposito and S. Cipriani	Biomedical Reports	English	Reviews (Literature, Narrative reviews, Systematic Reviews, Meta analysis)	ambulatory / outpatient	Italy	7 studies (1 of interest)	clinical studies in English, studies reporting original data, studies reporting the number of patients using D mannose alone or in association with other treatments, studies reporting number of patients with symptoms of UTI/cystitis at trial entry and after the follow up period.	to perform a systematic review of the available data on the effect of D mannose on the typical symptoms of UTI/cystitis given alone or in association with other compounds	Phytotherapy vs. Antibiotics	Burden of symptoms
2024	Non-steroidal anti-inflammatory drugs for treating symptomatic uncomplicated urinary tract infections in non-pregnant adult women	A Sachdeva, B. P. Rai, R. Veeratterapillay, C. Harding and A. Nambiar	Cochrane Database of Systematic Reviews	English	Reviews (Literature, Narrative reviews, Systematic Reviews, Meta analysis)	ambulatory / outpatient	UK	6 studies	RCTs and quasi RCTs looking at the effectiveness of NSAIDs in the treatment of symptomatic uncomplicated UTIs in non-pregnant adult women	to investigate the benefits and risks associated with the use of NSAIDs in the treatment of symptomatic uncomplicated UTIs in non-pregnant adult women	NSAIDs vs. Antibiotics	Symptom resolution<br>SAE/AE<br>Clinical resolution<br>Secondary antibiotic treatments<br>AB consumption (total number of courses)

2015	Urinary tract infection	N. Sheerin	Medicine	English	Reviews (Literature, Narrative reviews, Systematic Reviews, Meta analysis)	ambulatory / outpatient	UK	n.a.	evidence of UTI	to review urinary tract infections	ASPT vs Antibiotics	AB consumption (total number of courses)
2019	Urinary tract infection	N. Sheerin and E. Glover	Medicine	English	Reviews (Literature, Narrative reviews, Systematic Reviews, Meta analysis)	ambulatory / outpatient	UK	n.a.	evidence of UTI	to review urinary tract infections	ASPT vs Antibiotics	ASPT are suggested
2013	Unambiguous practice guidelines on urinary tract infections in primary and secondary care	K. M. van Asselt, J. M. Prins, G. M. van der Weele, B. J. Knottnerus, B. van Pinxteren and S. E. Geerlings	Nederlands Tijdschrift voor Geneeskunde	Dutch	Guidelines / Best practice / Commentary	ambulatory / outpatient	Netherlands	n.a.	evidence of UTI	to describe the dutch guidelines in UTI	ASPT vs Antibiotics	ASPT are suggested
2011	Qualitative interview study: managing urinary tract infections	A. Walker	BMJ	English	Guidelines / Best practice / Commentary	GP / PC	UK	1 study	Leydon 2010 study	to present Leydon 2010 research	delayed AB vs immediate AB	Alternative to antibiotics are welcomed
2017	Antibiotics or NSAIDs for uncomplicated urinary tract<br>infection?	P. Little	Bmj	English	Guidelines / Best practice / Commentary	ambulatory / outpatient	UK	n.a.	evidence of uUTI	to discuss NSAIDs and delay AB for uUTI	ASPT vs Antibiotics	Burden of symptoms<br>PN<br>ASPT are suggested
2024	Prediction of antibiotic prescription for acute uncomplicated cystitis: Insights from two randomized clinical trials	J. F. Alidjanov, S. Hoch, H. Steindl, D. Abramov-Sommariva, M. Höller, V. Wimmelbacher, K. Naber, F. M. E. Wagenlehner and C. Abels	European Urology	English	Abstract	ambulatory / outpatient	Germany	454	female patients (mean age: 44.0 years; SD = 15.5)	to identify the factors contributing to antibiotic prescription in female patients receiving non-antimicrobial therapy for AC	BNO 1045 (X 7days) and follow-up of 31 days	Secondary antibiotic treatments<br>Factors associated with secondary AB
2019	Exploring Maltese women's experiences of urinary tract infection: A qualitative study	E. Cutajar, K. Currie, P. Flowers and A. Dickson	Antimicrobial Resistance and Infection Control	English	Abstract	ambulatory / outpatient	Malta	19	women	to better understand the views and drivers of behaviours in relation to the management of UTI in Maltese women	ASPT vs Antibiotics	General aversion towards antibiotics as well as a relative openness to alternative methods of managing UTI
2009	Dipsticks and diagnostic algorithms in urinary tract infection: development and validation, randomised trial,	P. Little, S. Turner, K. Rumsby, G. Warner, M. Moore, J. A. Lowes, H. Smith, C. Hawke, D.	Health Technology Assessment	English	Report / Monograph	ambulatory / outpatient	UK	6 studies	report of selected evidence of women aged 17–70 with	to compare management using clinical and dipstick scores with commonly used alternative strategies; to estimate the cost-effectiveness of each strategy; and to	five management approaches: empirical antibiotics; empirical delayed antibiotics; target antibiotics based on a higher symptom score; target antibiotics based on dipstick results; or	Symptom resolution<br>AB consumption (total number of courses)<br>Burden of symptoms<br>Re-consultation<br>Costs<br>Positive view about delay AB<br>Not feeling considered / not taken seriously<br>Feeling that it's too late to delay when consulting

	economic analysis, observational cohort and qualitative study	Turner, G. M. Leydon, A. Arscott and M. Mullee	Assessment						17–70 with suspected UTI	understand the natural history of UTI and women's concerns about its presentation and management.	based on dipstick results, or target antibiotics based on a positive midstream specimen of urine (MSU)	Patients need to understand the rationale for delay<br>Fear of progression or worsening of symptoms<br>Prior positive experience / satisfaction with AB treatment<br>Avoiding side effects of AB<br>Delayed AB as a security (in case of emergency or when tryng alternatives)
2015	Uncomplicated Urinary Tract Infection in Primary Care : Evaluation of Point of Care Tests and Patient Management	E. Bongard	Cardiff University (United Kingdom)	English	Dissertation / thesis	GP / PC	UK	n.a.	narrative systematic review of observational studies of the management of uUTI; presenting results of a RCT on point of care test (POCT)	to describe the management of uncomplicated urinary tract infection (UTI) in primary care; to evaluate factors associated with different management decisions	ASPT vs Antibiotics	Willing to delay antibiotics<br>Positive view about delay AB<br>Clinical resolution<br>AB consumption (total number of courses)<br>Delayed prescriptions and use of analgesic
2020	Suplementos Alimentares na Prevenção e Tratamento de Infeções Urinárias	B. de Freitas Pina Setoca	Egas Moniz School of Health and Science (Portugal);	Portoguese	Dissertation / thesis	ambulatory / outpatient	Portugal	n.a.	bibliographical review; survey to pharmacists on dispensing	to find out more about the preventive and therapeutic approach to urinary infections, using food supplements.	ASPT vs Antibiotics	AB consumption (total number of courses)<br>Evidence of herbal extracts is scarce / inadequate<br>Symptom resolution<br>Safety<br>SAE/AE
2018	A Fitoterapia na Profilaxia e Terapêutica de Infeções do Trato Urinário não Complicadas: O Caso Particular da Cistite	A. C. M. Fraqueza	Universidade do Algarve (Portugal)	Portoguese	Dissertation / thesis	pharma	Portugal	n.a.	bibliographical review; online survey to pharmacists on recommending herbal products and patient satisfaction	to gather information on the products (herbal, food supplements) most commonly used in clinical practice for UTI.	Phytotherapy vs. Antibiotics	Recommendation of the herbal products for patients with UTIs<br>Patients ask for AB: they only look for herbal products when referred to them by a health professional<br>There is no substitute for AB<br>Feedback on user satisfaction of herbal products is positive
2021	Cranberry Extract for Symptoms of Acute, Uncomplicated Urinary Tract Infection	O. Gbinigie	University of Oxford (United Kingdom)	English	Dissertation / thesis	ambulatory / outpatient	UK	n.a.	review and RCT	to identify and appraise the existing evidence base for using cranberry extract as a treatment for symptoms of acute, uncomplicated UTI; to establish the views of UTI patients on using cranberry extract for symptoms of acute UTI	ASPT vs Antibiotics	AB consumption (total number of courses)<br>Burden of symptoms<br>Symptom resolution<br>SAE/AE<br>PN<br>Secondary antibiotic treatments<br>Evidence of herbal extracts is scarce / inadequate<br>Delayed is more acettable if combined with something patients perceive as therapeutical<br>Willing to delay antibiotics<br>Factors associated with ASPT<br>Cultural differences in the way that UTIs and antibiotics are perceived.
												Faster / immediate symptom relief taking AB<br>First trying alternatives and then if not working ask for antibiotics<br>A delay of AB is acceptable in case of mild symptoms<br>Side effects of AB (thrush) less acceptable than cystitis

2012	Urinary tract infection in women aged 18-64: doctors', patients', and lay perceptions and understandings	J. H. Larcombe	University of Durham (United Kingdom)	English	Dissertation / thesis	ambulatory / pharma	UK	18	women aged 18-64	to discover women's ideas and beliefs regarding cystitis, its symptoms, management;	ASPT vs Antibiotics	Complementary medicine considered as supplementary medicine and better for prevention than cure
												Complementary medicines are used more for chronic problems, rather than acute relief
												There is no substitute for AB
												Need of reassurance
												Delay and seek help when symptoms are severe is common
												Holidays or travel away from home for weeks lead to ask for AB "just in case"
												Free prescriptions may encourage use of AB even if delayed
								26	medical professionals (including pharma, nurses)	to elicit the ideas and beliefs of medical professionals regarding the diagnosis and clinical management of UTI in adult women aged 18-64,		Medical school influences the management of UTI
												Guidelines can change behaviour
												Guidelines are less effective in experienced professionals
												Therapeutics courses changed doctors perceptions on the use of antibiotics (less use)
												Rarely promoting self-help treatments.
												Doctors' (in)tolerance of uncertainty significantly affects prescribing behaviour:
												Pharmacists and nurses are more protocol driven
												Patients expect to get AB
												Delay AB did not feature as a strategy (unless investigations are not available)
												The extension in routine opening hours increases access and prescriptions
												It is more likely to give empirical tratments in the afternoon or before holidays
												Women's expectations are a strong influence on UTI management
												Patients aren't as keen to accept antibiotics as in the past - more skeptical (but this manifests as increased self-treatment rather than reduced expectation of AB)
												Pharmaceutical companies no longer have a significant influence on antibiotic prescribing: no commercial advantages

2021	Plantas Medicinais: Fitoterapia nas Infecções do Trato Urinário	L. C. Nunes	Universidade de Lisboa	Portoguese	Dissertation / thesis	ambulatory / outpatient	Portugal	n.a.	bibliographic review	to understand the importance of phytotherapy and to approach medicinal plants in the context of the prevention and treatment of urinary infections as an alternative or complement to conventional therapy	Phytotherapy vs. Antibiotics	AB consumption (total number of courses)
2021	The Feasibility of Using Electronic Health Records to Inform Clinical Decision Making for Community-Onset Urinary Tract Infection in England	P. Rockenschaub	University of London, University College London (United Kingdom)	English	Dissertation / thesis	GP / PC	UK	650,416	women	to estimate the risk of infectious complications associated with delaying or withholding antibiotics in adult women presenting with community-onset lower UTI in English primary care	delayed AB vs immediate AB	Factors associated with progressing to severe UTI
												Factors associated with all-cause mortality
2017	Alternative Treatments for Female Urinary Tract Infections : Microbiological Analysis of Herbal Medicinal Product, and Qualitative Study into Patients' Perspectives	J. Trill	University of Southampton (United Kingdom)	English	Dissertation / thesis	ambulatory / outpatient	UK			reviews and pharmacologic consideration	ASPT vs Antibiotics	ASPT are suggested
								20	women (included also > 50 years) the majority were from ATAFUTI see Moore 2019	to explore and evaluate patients' views and opinions towards delayed prescribing of antibiotics for urinary tract infections in favour of an alternative symptom treatment; to identify any barriers towards the prescribing of herbal medicine for UTI;	ASPT vs Antibiotics	Expectation that an antibiotic would be prescribed
												Expectation that the antibiotic will work
												The need for symptom relief prevails over fear of resistance
												First trying alternatives and then if not working ask for antibiotics
												Delayed prescription facilitates participation to trials investigating non AB treatment
												Side effects of AB are of great concern
												Cranberries and other remedies help but don't clear the symptoms
												Concern about the taste of cranberry juice, and particularly the sugar content
												Herbal medicines are not considered as medicines but as plants
												Availability of herbal medicine OTC save doctor's appointment
2012	Uncomplicated urinary tract infections in general practice.	B. J. Knottnerus	University of Amsterdam	English	Dissertation / thesis	GP / PC	Netherlands	176	women aged 16-89 yrs otherwise healthy not pregnant contacting the GP for symptoms of UTI lasting no longer than 7 days	to focus on UTI in general practice.	delayed AB asked (137 patients) vs. not asked	Willing to delay antibiotics
												Clinical resolution
												PN
												Symptom resolution
												AB consumption (total number of courses)
												Factors associated with ASPT

2020	Non-interventional study with Femannose® N to investigate tolerance, quality of life and course of symptoms in acute uncomplicated urinary tract infection	F. M. Wagenlehner, L. N. Baumgartner, B. Schopf and J. Milde	J. Pharmakol Ther.	German	Cohort study (prospective, retrospective)	ambulatory / outpatient	Germany	103	women aged≥18 years with an acute uUTI	to generate clinical evidence of the use and the potential benefit and the risks of D-mannose	D-mannose (Femannose® N) monotherapy (29%) vs. D-mannose in combination with antibiotics (55%) vs. the combination of D-mannose with other therapeutic measures (16%) - treatment duration varied between 3 and 7 days	Symptom resolution
												Patient satisfaction
												Safety

Supplemental table 10: Preferred Reporting Items for Systematic reviews and Meta-Analyses extension for Scoping Reviews (PRISMA-ScR) Checklist

SECTION	ITEM	PRISMA-ScR CHECKLIST ITEM	REPORTED ON PAGE #
**TITLE**			
Title	1	Identify the report as a scoping review.	1
**ABSTRACT**			
Structured summary	2	Provide a structured summary that includes (as applicable): background, objectives, eligibility criteria, sources of evidence, charting methods, results, and conclusions that relate to the review questions and objectives.	2
**INTRODUCTION**			
Rationale	3	Describe the rationale for the review in the context of what is already known. Explain why the review questions/objectives lend themselves to a scoping review approach.	3
Objectives	4	Provide an explicit statement of the questions and objectives being addressed with reference to their key elements (e.g., population or participants, concepts, and context) or other relevant key elements used to conceptualize the review questions and/or objectives.	4
**METHODS**			
Protocol and registration	5	Indicate whether a review protocol exists; state if and where it can be accessed (e.g., a Web address); and if available, provide registration information, including the registration number.	4
Eligibility criteria	6	Specify characteristics of the sources of evidence used as eligibility criteria (e.g., years considered, language, and publication status), and provide a rationale.	4
Information sources*	7	Describe all information sources in the search (e.g., databases with dates of coverage and contact with authors to identify additional sources), as well as the date the most recent search was executed.	4
Search	8	Present the full electronic search strategy for at least 1 database, including any limits used, such that it could be repeated.	supplemental
Selection of sources of evidence†	9	State the process for selecting sources of evidence (i.e., screening and eligibility) included in the scoping review.	4
Data charting process‡	10	Describe the methods of charting data from the included sources of evidence (e.g., calibrated forms or forms that have been tested by the team before their use, and whether data charting was done independently or in duplicate) and any processes for obtaining and confirming data from investigators.	4, 5
Data items	11	List and define all variables for which data were sought and any assumptions and simplifications made.	supplemental

SECTION	ITEM	PRISMA-ScR CHECKLIST ITEM	REPORTED ON PAGE #
Critical appraisal of individual sources of evidence§	12	If done, provide a rationale for conducting a critical appraisal of included sources of evidence; describe the methods used and how this information was used in any data synthesis (if appropriate).	n.a.
Synthesis of results	13	Describe the methods of handling and summarizing the data that were charted.	4,5
**RESULTS**			
Selection of sources of evidence	14	Give numbers of sources of evidence screened, assessed for eligibility, and included in the review, with reasons for exclusions at each stage, ideally using a flow diagram.	Figure 1
Characteristics of sources of evidence	15	For each source of evidence, present characteristics for which data were charted and provide the citations.	6, table1, supplemental
Critical appraisal within sources of evidence	16	If done, present data on critical appraisal of included sources of evidence (see item 12).	n.a.
Results of individual sources of evidence	17	For each included source of evidence, present the relevant data that were charted that relate to the review questions and objectives.	supplemental
Synthesis of results	18	Summarize and/or present the charting results as they relate to the review questions and objectives.	6, 7
**DISCUSSION**			
Summary of evidence	19	Summarize the main results (including an overview of concepts, themes, and types of evidence available), link to the review questions and objectives, and consider the relevance to key groups.	8
Limitations	20	Discuss the limitations of the scoping review process.	9
Conclusions	21	Provide a general interpretation of the results with respect to the review questions and objectives, as well as potential implications and/or next steps.	9
**FUNDING**			
Funding	22	Describe sources of funding for the included sources of evidence, as well as sources of funding for the scoping review. Describe the role of the funders of the scoping review.	10

JBI = Joanna Briggs Institute; PRISMA-ScR = Preferred Reporting Items for Systematic reviews and Meta-Analyses extension for Scoping Reviews.
* Where *sources of evidence* (see second footnote) are compiled from, such as bibliographic databases, social media platforms, and Web sites.
† A more inclusive/heterogeneous term used to account for the different types of evidence or data sources (e.g., quantitative and/or qualitative research, expert opinion, and policy documents) that may be eligible in a scoping review as opposed to only studies. This is not to be confused with *information sources* (see first footnote).
‡ The frameworks by Arksey and O'Malley (6) and Levac and colleagues (7) and the JBI guidance (4, 5) refer to the process of data extraction in a scoping review as data charting.
§ The process of systematically examining research evidence to assess its validity, results, and relevance before using it to inform a decision. This term is used for items 12 and 19 instead of "risk of bias" (which is more applicable to systematic reviews of interventions) to include and acknowledge the various sources of evidence that may be used in a scoping review (e.g., quantitative and/or qualitative research, expert opinion, and policy document).
From: Tricco AC, Lillie E, Zarin W, O'Brien KK, Colquhoun H, Levac D, et al. PRISMA Extension for Scoping Reviews (PRISMAScR): Checklist and Explanation. Ann Intern Med. 2018;169:467–473. doi: 10.7326/M18-0850.
